# Supplementary material for: Adapted “Break the Cycle for Avant Garde” intervention to reduce injection assisting and promoting behaviours in people who inject drugs in Tallinn, Estonia: A pre- post trial
Source: PLoS One. 2023 May 31;18(5):e0266815. doi: 10.1371/journal.pone.0266815 (PMC10231841; doi:10.1371/journal.pone.0266815)
Supplement: S1 Protocol — (PDF) [file pone.0266815.s002.pdf]

UURIMISTÖÖ AVALDUS KOOSKÕLASTUSE SAAMISEKS TARTU ÜLIKOOLI INIMUURINGUTE EETIKA KOMITEELT

Combined prevention for reducing initiation into injecting drug use.

Sponsored by: National Institutes of Health

NIH Funding Mechanism: Grant #1DP1DA039542

Protocol Chair:

Don Des Jarlais, PhD
Beth Israel Medical Center
160 Water Street, Rm 2462
New York NY 10038
Phone +1 212 256 2548
Fax +1 212 256 2570
ddesjarlais@chpnet.org

Protocol Co-Chair:

Anneli Uusküla, MD, MS, PhD
University of Tartu, Department of Public health
Ravila 19, Tartu 50411, Estonia
Phone + 3727374195
Fax + 3727374192
Email anneli.uuskula@ut.ee

Study Coordinator:

Ave Talu, MS
University of Tartu, Department of Public health
Ravila 19, Tartu 50411, Estonia
Phone + 3727374195
Fax + 3727374192
Email marsh.kristina@gmail.com

Statistician
Mait Raag, MS
University of Tartu, Department of Public health
Ravila 19, Tartu 50411, Estonia
Phone + 3727374201
Fax + 3727374192
Email mait.raag@ut.ee

Study Sites:

University of Tartu
Department of Public Health
Ravila 19, Tartu 50411, Estonia

NGO Convictus
Syringe exchange program
Lastekodu 6, Tallinn 10113, Estonia

1. UURIMISTÖÖ ANDMED

Uurimistöö täielik nimetus: Kombineeritud sekkumine süstimisega alustamise vähendamiseks.

Inglise keeles: Combined prevention for reducing initiation into injecting drug use.

Uurimistöö toimumiskoht:

TÜ peremeditsiini ja tervishoiu instituut, Ravila 19, Tartu 50411
MTÜ Convictus, Lastekodu 6, Tallinn 10115

2. UURIMISTÖÖ TEOSTAJAD JA UURINGUKESKUSED

2.1. Vastutav uurija
ees- ja perekonnanimi: Anneli Uusküla
teaduslik kraad: MD, MS, PhD
amet: professor
töökoht: Tartu Ülikool, peremeditsiini ja rahvatervishoiu instituut
töökoha aadress: Ravila 19, Tartu 50409
telefoninumber: 737 4195
e-post: anneli.uuskula@ut.ee
kuupäev: allkiri:

2.2. Kaastöötajad
ees- ja perekonnanimi: Ave Talu
teaduslik kraad: MSc
amet: projektijuht
töökoht: TÜ peremeditsiini ja rahvatervishoiu instituut
töökoha aadress: Lastekodu 3, Tallinn 10115
e-post: ave.talu@gmail.com
kuupäev: allkiri:

ees- ja perekonnanimi: Greete Org
amet: superviisor
töökoht: TÜ peremeditsiini ja rahvatervishoiu instituut
töökoha aadress: Lastekodu 6, Tallinn 10115
e-post: greete.org@mail.ee
kuupäev: allkiri:

2.3.Vastutava uurija asutuse juhataja või tema kohusetäitja
on uurimistöö korraldamisega nõus:
ees- ja perekonnanimi: Ruth Kalda
amet: peremeditsiini ja rahvatervishoiu instituudi juhataja
töökoht: Tartu Ülikool
töökoha aadress: Ravila 19, Tartu 50409
kuupäev: allkiri:

2.4. Uurimistööga kaasatud muu asutuse kooskõlastus:
volitatud isiku ees-ja perekonnanimi: Inna Faber
amet: tegevjuht
töökoht: MTÜ Convictus Eesti
töökoha aadress: Lastekodu 6, Tallinn, 10113
kuupäev: allkiri:

3. UURIMISTÖÖ FINANTSEERIMINE

Allikas: Uurimustöö finantseerijaks on National Institutes of Health (USA) Grant Number DA039542 (TÜ finantsallikas TMVPT15017I).

Uuritavatele kompensatsiooni maksmine:
Uuritavatele isikutele makstakse kompensatsiooni uuringus osalemisele kulunud aja ja võimalike transpordi kulude katteks: igale uuritavale on uuringu igal visiidil (kaasamisvisiit, järelvisiit) osalemise eest ette nähtud kompensatsiooniks kinkekaart 20 EUR väärtuses (RIMI kaupluse kinkekaart)

Iga uuritava poolt uuringusse suunatud mitte-süstivast, süstivast või kunagi süstinud, kuid viimasel kahel kuul mitte-süstinud narkootikume tarvitava sõbra / tuttava eest (maksimaalselt 3 isikut) saavad uuritavad (peale uue uuringusse suunatu uuringusse kaasamist) uuringupreemiaks kinkekaardi väärtuses 10 eurot (RIMI kaupluse kinkekaart).

Uuritavad ei ole uuringuga seoses kindlustatud.

4. LÜHIÜLEVAADE SIIANI SAMAL TEEMAL LÄBIVIIDUD UURIMISTÖÖDEST

Hinnanguliselt on ca 1% (5362 inimest; UI 3906– 9837) Eesti rahvastikust vanuses 15–44 aastat süstivad narkomaanid (Uusküla et al., 2013). On leitud, et narkootikumide süstimisega alustatakse Eestis väga noorelt. 23% Eestis uuritud süstivatest narkomaanidest alustas narkootikumide tarvitamist nooremalt kui 15-aastaselt ja nende hulgas oli HIV levimus kõrgem kui hilisemas vanuses süstimist alustanutel (48% versus 68%) (Vorobjov et al., 2013). Eestis narkootikumide süstijate uuringud on näidanud, et pooled süstijatest on alustanud narkootikumide kasutamist kohe süstides, teine pool süstijatest on enne süstimise alustamist kasutanud narkootikume mõnel teisel moel (suu/ninakaudu, suitsetades jm) (Vorobjov 2012; 2013).

Enamikes Euroopa Liidu liikmesriikides on vähenenud uute HIV-juhtude registreerimine narkootikumide süstijate hulgas, kuid Eesti on jätkuvalt nende riikide hulgas, kus uutest HIVi juhtudest veerand kuni kolmandik (Eesti 25%, Luksemburg 27%, Läti 32% ja Leedu 34%) registreeritakse narkootikume süstivate inimeste hulgas (EMCDDA, 2017).

Narkootikumide tarvitamist, süstimist ja süstimise alustamist on Eestis püütud vähendada informeerides inimesi narkootikumidega seotud ohtudest, seadusandlike meetmete (tarvitajate karistamine tarvitamise eest – rahatrahvi, vanglakarituse määramisega) ning ulatuslike politseioperatsioonide abil lisaks narkootikumide kõrvaldamisele illegaalselt narkoturult ja narkootikumidega kaubitsemisega seotud kuritegude eest pikaajaliste vanglakaristuse määramisele. Narkootikumidega seotud süüteod (NPLAS § 151 st väikeses koguses narkootilise aine tarvitamine, valmistamine, omandamine või valdamine) on järsult tõusnud (2014. a – 2862, 2015. a . 3633, 2016. a – 4372) kuna politsei viimaste aastate prioriteediks on narkootikumide tarvitajatele suunatud tegevus (TAI, 2017). Keskmine narkoväärteo (NPLAS § 151) eest narkosõltlastele määratud rahatrahv Eestis on ca 150 eurot, millele lisanduvad ekspertiisikulud.

Narkootikumide süstimine (kasutatud süstla jagamine) on HIVi ja C-hepatiidi epideemiate peamine põhjus kogu maailmas. See on ka oluline muu haigestumise (mitte surmaga lõppenud üleannustamine,

enesetapukatsed, naha- ja pehmete kudede infektsioonid) ja suremuse põhjus. .On leitud, et jõustruktuuride poolt kasutatav hirmutamine ja ülekriminaliseerimine olukorra parandamiseks ei tööta ning alternatiivsed strateegiad on vajalikud (Godlee F., 2018)
Rohkem kui 90 USA suurlinna andmetel (1992–2002) põhinev analüüs kinnitas, et narkootikumide tarvitamise eest määratud arestid ei vähendanud narkootikumide süstijate arvu ja tõhusamaks meetmeks on neile narkomaaniaravi võimaldamine (Friedman et al., 2011). Portugalis narkootikumide dekriminaliseerimise (sõltlaste karistamise asemel nende teenustele suunamine) vähendas narkosõltuvusega seotud stigma, narkootikumide tarvitamist ja narkootikumidega seotud surmajuhtumeid ja suurendas sõltlaste hõlmatust nõustamise, ravi- ja rehabilitatsiooniteenustega (McCaffrey, 2009) ning vähendas narkootikumidega seotud tervise ja mitte-tervisega seotud sotsiaalset kulu 18% (Gonçalves et al., 2015). 2018. aastal on tekkinud ka Eesti eri ametkondade (sh Justiitsministeerium, Siseministeerium, Sotsiaalministeerium, TAI jms) vahel arusaam, et trahvide ja menetluskulude tasumine käib sõltlasele üle jõu ja trahvimisega sõltuvuse ravimine (narkootikumidega seotud psüühika-ja käitumishäire on rahvusvahelise haiguste klassifikatsiooni järgi haigus) ei tööta. Alustati narkosõltlaste tugiisiku teenust (narkoväärteo eest karistuse asendamine ja sõltlase sidumine määratletud teenustega) pakkuvate projektidega arestimajas (Balti Kriminaalpreventsiooni Instituudi ja arestimajad) ja kainestusmajades (TAI ja Eesti Politsei koostööprojekt).

Narkootikumide süstimist kas ennetavad, või siis edasi lükkavad sekkumised on olulised, kuidtõendust selles olulises valdkonnas on vähe. Narkootikumide süstimist ennetavad sekkumised võivad olla suunatud: (i) narkootikumide süstijatele; või (ii) narkootikumide muul viisil tarvitajatele, kes süstinud ei ole ja vähese süstimiskogemusega inimestel, kes tarvitavad narkootikume peamiselt muul viisil kui süstides.
Narkootikumide süstimisega alustamise ennetamiseks on testitud ja tõendatult efektiivsed vaid üksikud sekkumised (süstemaatiline ülevaade: Werb D et al., 2013; Werb et al, 2018). On leitud, et süstimisega alustamist aitavad efektiivselt vähendada “Muuda tsüklit” (*ingl* Break the Cycle) (Hunt N et al., 1998) ja heroiini mitte süstitavalt („nuusutades“) kasutajatele suunatud projektid (Heroin Sniffers Project) (Des Jarlais et al., 1996). Kõik need sekkumised sisaldavad sotsiaalse võrgustiku ja sotsiaalse toetuse komponenti ja on suunatud süstimisega seotud sotsiaalsete normide muutmisele.

Illegaalsete narkootikumide süstimine on keeruline ja sageli ohtlik protseduur ning peaaegu kõik, kes alustavad süstimist, vajavad kogenud süstija abi enda esmakordseks süstimisel. Pikaajalise süstimise kogemusega süstijad on oluline ennetuse sihtrühm, kuna neil on oskused ja sageli ka võimalus varem mitte-süstinud isikuid esimese narko-süsti tegemisel abistada.

2016. aastal Tallinna narkootikumide süstijate (n=299) hulgas läbi viidud sekkumisuuringus “Katkesta ahel” (TÜ inimuuringute eetika komitee protokoll number 261/M-29, 22.08.2016) osalenutest 46% olid nakatunud HIVi, neist 66% olid antiretroviirusravil (Uusküla et al., 2018). Uuringus osalenutest viiendiku (20%) poole oli viimasel 6 kuul pöördutud palvega aitada esimese süsti tegemisel. Osalenutest 14% aidanud kedagi esmakordse narkosüsti tegemisel (4.3% olid seda teinud ka viimasel 6 kuul); viimase kuue kuu jooksul oli uuritavates 9% rääkinud positiivselt narkootikumide süstimisest (parem ja kiirem mõju ning majanduslikult tasuvam), 16% oli ennast süstinud mitte süstiva inimese ees ja 1.0% pakkunud abi mitte süstijale esmakordsel süstimisel (Uusküla et al., 2018). Mitte süstijale esmakordselt narkootikumide süstimine oli sagedasem meeste (AOR 6.31, 95% CI 2.02—19.74), nooremate (30-aastased ja nooremad) (AOR 3.89, 95% CI 1.40—10.16) ja nende hulgas, kel oli sõpru/tuttavaid, kes samuti olid abistanud teisi esmakordse narkosüsti tegemisl olemasoluga (AOR 3.44,95% CI 1.31—9.03) (Uusküla et al., 2018). Viimasel korral, kui süstijaid aitasid esimese narkosüsti tegemisel, oli see inimene neile tuttav (42%), sõber (35%), seksuaalpartner (13%) või võõras (8%). Peamiseks põhjuseks mitte-süstija abistamisel esimese narkosüsti tegemisel oli enda esimest korda süstida soovija vastavate oskuste puudumine (78%), tema seisund (liiga närviline ja käte värin segas süstimist) ei võimaldanud seda (46%) või ta ise palus ennast süstida (44%). Mitte-süstija süstimise eest jagasid nad oma narkootikumi süstijaga (33%), maksid talle (2%) ning 21% süstijatest soovis oma kogemust jagada (Uusküla et al., 2018). Uuringu „Katkesta ahel“ tulemused (hinnatuna 6 kuud peale sekkumist) näitasid sekkumise selget positiivset mõju.
Esimse visiidi ja järelvisiidi andmed näitasid süstimist propageeriva ja abistava käitumise märkimisväärset vähenemist: 68,8% (42/61st) uuriavatest, kes olid süstinud mitte-süstija ees esmakordselt visiidil, ütlesid kuue kuu järel toimunud järelvisiidil, et nad ei olnud seda teinud ja

90,9% (10/11) nendest, kes olid abistanud mitte-süstijat nende esimese süstiga 6 kuud enne uuringus osalemist, teatasid (süstimisel) mitte abistamisest järelvisiidil 6 kuud hiljem. Olulise leiuna selgus, et 7,8% osalenud süstijatest ei süstinud enam, kui neid küsitleti 6 kuud peale sekkumist (Uusküla et al. Töös olev käsikiri).
Mujal arenenud riikides hiljuti tehtud uuringute järgi oli 14% (Hamida et al., 2018) kuni 47% (Bluthenthal et al., 2014) narkootikumide süstijatest aidanud elu jooksul mõnel mitte-süstijal esmakordsel narkootikume süstiida. Kanadas läbi viidud uuring näitas, elu jooksul oli süstija abistanud keskmiselt 16-le inimesele esmakordsel narkootikume süstida (Bluedenthal et al, 2014). Kvalitatiivuuringutel põhinev meta-analüüs (n=41), et süstimisega alustamisel on einev tähendus inimestele ja see on seotud sotsiaalsete ja strukturaalsete teguritega, mistõttu lisaks sekkumistega saavutatava käitumise muutumisele lisaks tuleks keskenduda ka eelnimetatud teguritele (Guise et al., 2017).

Üleminek mitte-süstivalt narkootikumide tarvitamiselt süstivale kasutamisele ei ole vältimatu. On kirjeldatud erinevaid individuaalseid ja sotsiaalse-võrgutiku põhiseid tegureid, mis seda soodustavad (kodutus, töötus, noorem iga opioidide kasutamise alustamisel, süstivast narkomaanist tuttavate/sõprade/seksuaalpartneri olemasolu, tunnetatud sõprade või tuttavate toetus süstivale kasutamisele, süstimisel joobe saamiseks vajalik väiksem narkootikumi kogus) (Germade M et al., 2007).

Tallinnas on käigus uuring „Väldi nõela“ (TÜ inimuuringute eetika komitee protokoll number protokoll number 272/T-3, 21.08.2017), mille eesmärgiks on süstimisega alustamise vältimine või edasilükkamine ning, mis on suunatud narkootikume mitte-süstijatele või väga vähese süstimise kogemusega narkomaanidele. Käigusoleva uuringu järelvisiidid, 6 kuud peale kaasamist, lõppevad plaanikohaselt novembris 2018.a. Lähtudes seni kogunenud tõendusest on selge, et sekkumine “Väldi nõela” sekkumine sobis uuritavatele, kuid erinevatel põhjustel (sh hirm politsei ees, kartus isikustatud uuringus osaleda jms) osales uuringus vähe uuritavaid. Olulisim probleem osaluseks (kartus politsei ees) on lahenenud, kuna politsei uus suund alates maist 2018.a. on narkootikumide tarvitajatele karistuse asemel mittekaristuslike meetmete pakkumine (nõustamine, ravi ja rehabilitatsioon). Eelnimetatud uuringu vältel kujunes meil ka hea koostöö erinevate asutustega (Siseministeerium, Justiitsministeerium, Politsei- ja Piirivalveamet, Põhja Politseiprefektuuri Arestimaja Tallinnas, Eesti psühhotroopsete ainete sõltlaste ühing LUNEST, TAI, Tallinna Wismari Haigla, Regionaalhaigla psühhiaatriakliiniku kaksikdiagnoosiga patsientide päevaravi osakond ja Psühhiaatriapolikliinik, Lääne- Tallinna Keskhaigla Psühhiaatriakeskus, Tallinna Lasnamäe Mehhaanikakool, Ida-Harju Kriminaalhooldusosakond, Lääne-Harju Kriminaalhooldusosakond, MTÜ Convictus Eesti, MTÜ Pealinna Abikeskus) uuritavate värbamiseks.

Narkootikume süstivate inimeste arvu ja uute süstijate juurdekasvu pidurdamiseks seni Eestis kasutatud repressiivsed meetmed ei soodusta abivajajate seotust tervishoiuteenustega (sh narkomaaniaravi ja antiretroviirusraviga) ja muude teenustega (sh üledoosi surmasid ennetav naloksooni programm).

5. KAVANDATAVA UURIMISTÖÖ EESMÄRK, KOKKUVÕTE JA PÕHJENDUS

Uuringu põhieesmärgiks on vähendada narkootikumidega süstimise alustamist toimides paralleelselt kahele sihtrühmale: (1) süstivad narkomaanid ja (2) mitte-süstivad narkomaanid. Uuringus kasutatakse motiveerival intervjueerimisel põhinevaid sekkumisi “Katkesta ahel” ja „Muuda tsüklit“. Mõlemad sekkumised on kohandatud kasutamiseks sihrühmadele Eestis ning testitud varasemates uuringutes Tallinnas (“Katkesta ahel”: protokolli number: 261/M-29, 22.08.2016; „Väldi nõela“: protokolli number: 272/T-3, 21.08.2017). Lähtuvalt juba teostatud uuringutest on käesoleva projekti lisa eesmärk koguda täiendavat teõndust sekkumiste efektiivsuse osas ning andmeid mitte-süstivate ja süstivate narkomaanide sotsiaalsete/narkootikumide kasutamisega seotud võrgustike (kattuvuse) osas.

Uuringus kasutatakse motiveerival intervjueerimisel (MI) põhinevat sekkumist:
(i) narkootikumide süstijatele suunatud “Katkesta ahel”, milles arutatakse süstijatega, kuidas see

on nende elu mõjutanud, nende esimese süstimiskogemuse üle ja kellegi abistamisel esmakordse narkosüsti tegemisel ja muudest tegevustest (demonstreerimine, propageerimine), mis võivad mõjutada kedagi alustama süstimist jaRäägitakse süstimisega kaasnevatest ohtudest, situatsioonidest, mis võivad viia kellegi süstimiseni, teistele süstijatele teabe jagamiseni ning teiste süstimisega aitamise ohtude kohta. Lisaks tutvustatakse sekkumisel ohutut süstimist ja käsitletakse üledooseerimise riske ja nende ennetamise võimalusi.

(ii) "Väldi nõela", mille raames nõustatakse praeguseid mitte-süstivaid narkomaane (kes on süstimisele üleminekuks kõrge riskiga - fentanüüli kasutajad, amfetamiini ja kokaiini kasutaja) ära tundma olukordi, mis võiksid viia esimese narkosüstini, hoiduma sellistest olukordadest ning neis toimetuleku strateegiaid. "Väldi nõela" annab osalejale ka paremad teadmised ohutust süstimisest ja üledooside ennetamisest

(Sekkumise juhised taotlusele lisatud).

Mõlemad uuringus rakendatud sekkumised põhivad all esitatud teaduskirjandusel ning on kohandatud lähtuvalt 2016-2018 aastal Tallinnas läbi viidud sekkumisuuringutele ja arvestavad kohalikke olusid (enam kasutatud narkootikumid, ravi ja ennetusvõimalused).

1) Des Jarlais, D. C., Casriel, C., Friedman, S. R., & Rosenblum, A. AIDS and the transition to illicit drug injection – results of a randomized trial prevention program. British Journal of Addiction 1992;87:493-498.
2) Casriel C, Des Jarlais DC, Rodriguez R, Friedman SR, Stepherson B, Khuri E. Working with heroin sniffers: clinical issues in preventing drug injection. J Subst Abuse Treat. 1990;7(1):1-10.
3) Hunt, N., Stillwell, G., Taylor, C., Griffiths, P., 1998. Evaluation of a brief intervention to prevent initiation into injecting. Drugs Educ. Prev. Policy 5, 185–194.
4) Strike C, Rotondi M, Kolla G, Roy É, Rotondi NK, Rudzinski K, Balian R, Guimond T, Penn R, Silver RB, Millson M, Sirois K, Altenberg J, Hunt N. Interrupting the social processes linked with initiation of injection drug use: results from a pilot study. Drug Alcohol Depend. 2014 Apr 1;137:48-54.
5) Werb D, Buxton J, Shoveller J, Richardson C, Rowell G, Wood E. Interventions to prevent the initiation of injection drug use: A systematic review. Drug Alcohol Depend. 2013, Dec 133(2): 669-676.

Uuringus kasutatavate sekkumisest kohandamine on toimunud koostöös New Yorgi, Icahn School of Medicine at Mount Sinai ja Columbia Ülikooli teadlastega. Don Des Jarlais on sekkumise „Heroiini-nuusutajate projekt" väljatöötaja ja testija, Susan Tross praktiseeriv kliiniline psühholoog. Nii süstijatele kui ka mitte-süstijatele suunatud sekkumisi testitakse paralleelselt Tallinnas ja New Yorgis.

Planeeritud sekkumisel on potentsiaali vähendada narkootikumidega süstimise alustamist, sellega seotud HIVi nakatumist ja vähendada teisi süstimisega seotud riske üksikisikule ja ühiskonnale. Uuringu raames rakendatud sekkumine toetab ka üledooside ja narkosurmade ennetust, kuna sisaldab komponendina ka üledooside ennetavale sekkumisele suunamist. Plaanitud uuringu raames on hea võimalus sõltlasi informeerida professionaalse nõustamise, narkomaaniaravi ja kahjude vähendamise teenuste võimalustest (sh üledooside ja narkosurmade ennetamiseks mõeldud naloksooniprogramm). Tavaliselt satuvad uuritavate poolt juhitud kaasamise meetodil uuringusse palju isikuid, kes seni pole endale oma tarvitamisega seotud probleeme teadvustanud ning ei ole nõustamise, ravi ega muude teenustega seotud. Uuring loob võimalusi suunata neid neile sobivate teenuste juurde.

Uuringu põhieesmärgiks on vähendada narkootikumidega süstimise alustamist. Me eeldame, et sekkumise tulemusel väheneb osalejate hulgas süstimisega alustamine ja/või sellele suunatud tegevus. Uuringu küsimustele vastamiseks viime läbi eksperimentaalse uuringu, kus kaasatud narkomaane, kes on saanud nõustamise jälgitakse 6 kuud peale sekkumist.

6. UURIMISTÖÖ TEOSTAMISE AEG

Töö teostamine (uuritavate kaasamine, jälgimine ja andmete kogumine, HIVi ja C-hepatiidi suhtes testimine) on planeeritud perioodiks august 2018 – oktoober 2019.

Värbamisperiood kestab orienteeruvalt 6 kuud, mille vältel kaasatakse uuringusse kokku 300 narkootikumide tarvitajat (nii isikuid, kes süstivad narkootikume kui ka mitte-süstivat ja/või viimasel 2 kuul mitte süstinud, kuid varasema vähese süstimiskogemusega narkootikumide tarvitajat) Tallinnast ja Harjumaalt.

Uuringus osaleja jaoks kestab uuring kokku 26 nädalat, see on ajavahemik kaasamisvisiidist ja sellel osalemiseks informeeritud nõusoleku allkirjastamise päevast kuni teise põhivisiidini, antud ajavahemikus külastab osaleja uuringukeskust kaheks (narkootikumide süstija, kes osaleb "Katkesta ahel" sekkumisel) või kolmeks (mitte-süstija ja/:või viimasel 2 kuul mittesüstinud, kuid varasema vähese süstimiskogemusega narkootikumide tarvitaja, kes osaleb "Väldi nõela" sekkumisel) uuringu visiidiks.

Andmete sisestamise, sisestuse kontrolli ja analüüsiga alustatakse kohe peale uuritavate uuringus esmakordset osalemist 2018. aasta augustis.

Uuringusse kaasamise koht:

> Uuritavate värbamine uuringusse toimub Tallinnas TÜ uuringu tarbeks renditud ruumides (MTÜ Convictus Eesti, Lastekodu 6). Kahjude vähendamise teenuseid pakkuva MTÜ Convictus ruumid on sobivad uuringu läbiviimiseks – tsentraalse paigutusega Tallinnas, omaette sissekäiguga nii hoovist kui ka tänavalt; avar vastuvõtu tuba ja kaks privaatset ruumi intervjuude ning sekkumiste läbiviimiseks ning spetsiaalne eraldi ruum privaatselt uuritavate HIVi ja C-hepatiiti suhtes testimiseks.

Uurimistöö teostamise eelselt viiakse läbi järgmised koolitused:

1. uuringusse kaasamise ja andmete kogumise meeskonnale uuringu toimingute, uuringus isiku privaatsuse ja andmete konfidentsiaalsuse koolitus;
2. uuringu intervjueerijatele intervjueerimise koolitus;
3. HIVi ja C-hepatiidi testimise (testieelne ja testijärgne nõustamine) koolitus Synlabis;
4. uuringus osalevate sekkujate (nõustajate) nõustamiskoolitused:
    (i) sekkumise metoodika- motiveeriva intervjueerimise koolitus (EMITA - http://www.emita.ee/ - poolt).;
    (ii) sekkumise spetsiifiline koolitus – koolitajad D. Des Jarlais, D. Barnes (toimub juulis 2018)
5. uuringu sekkujad osalevad uuringu käigus regulaarselt EMITA supervisioonis.

Uuringus osalevad sekkujad töötavad sihtrühma nõustajatena oma igapäevases töös ja on osalenud sekkujatena varasemates uuringutes, mis eelpool kirjeldatud. Uuringus osalenud intervjueerijad on osalenud samuti nende uuringute tegemisel ja saanud ka eelneva väljaõppe intervjueerimiseks.

7. UURITAVATE JA NENDE VÄRBAMISVIISI TÄPNE KIRJELDUS

Käesolevas uuringusse värvatakse uuritavaid kasutades uuritavate poolt juhitud kaasamise meetodit (*ingl* respondent driven sampling), mida on varem Eesti uimastitarbijate uuringutes kasutatud (aastatel 2005 – 2017). RDS on kaasaegne ning valideeritud meetod uimasteid tarvitavate inimeste uuringusse haaramiseks (Malekinejad et al, 2008).

Uuritavate poolt juhitud kaasamise meetodi kohaselt valitakse esimesed uuritavad (käesolevas uuringus 10 uimastitarvitajat (nn "seemned"). Eesmärgiks on kaasata uuringusse võimalikult erinevad uimastitarvitajad (soo, rahvuse, vanuse, peamiselt kasutatava uimasti, HIVi staatuse lõikes)

saavutamaks uuringu valimi maksimaalne esinduslikkus (representatiivsus). Selle saavutamiseks otsime uuringuks sobivaid seemneid koostööpartnerite (sh Eesti psühhotroopsete ainete sõltlaste ühing LUNEST, Tallinna Wismari Haigla, Regionaalhaigla psühhiaatriakliiniku kaksikdiagnoosiga patsientide päevaravi osakond ja Psühhiaatriapolikliinik, Lääne- Tallinna Keskhaigla Psühhiaatriakeskus, Ida-Harju Kriminaalhooldusosakond, Lääne-Harju Kriminaalhooldusosakond, MTÜ Convictus Eesti, Põhja Politseiprefektuuri Arestimaja ja kainestusmaja Tallinnas, Tallinna Lasnamäe Mehhaanikakool, Tallinna Tööstushariduskeskus) kaasabil, kellega on meil kujunenud varasemate uuringut tegemisel hea koostöö. Uuringu kutse partner organisatsioonidele edastab projektijuht.

Uuringu käigus intervjueeritakse esmalt "seemneid" ja siis palutakse neil igaühel leida kolm uut uuringu kriteeriumitele vastavat uuritavat. Selleks antakse „seemnetele" kolm kupongi, millel on kirjas info uuringu kohta sh RDS kupongi number, uuringukeskuse telefoninumber, e-mail ning lahtioleku kellaajad ja koht, kus uuring läbi viiakse. "Seeme" annab saadud kolm kupongi järgmistele talle teadaolevatele uimastitarvitajatele (narkootikumide süstija või mitte-süstija).

Uuritavate kaasamisega seotud toimingud (sh uuringusse sobivuse selgitamine, uuringu protseduuride selgitamine, informeeritud nõusolek, uuringusse registreerimine, uuringvisiitide aegadest informeerimine) viiakse läbi uuringu keskuses superviisori (G. Org) või projektijuhi (A. Talu) poolt.

Uuringusse kaasamise kriteeriumid:

- 18-aastane ja vanem Tallinnas ja Harjumaal elavad nais- ja meessoost isik,
- võimeline andma informeeritud nõusoleku uuringus osalemiseks,
- on enda ütluse kohaselt kasutanud illegaalseid narkootikume (peamiseks tarvitatavaks narkootikumiks kas fentanüüli või muid opiaate, amfetamiini, metamfetamiini, ecstacy't, va marihuaana) viimase 2 kuu jooksul,
- räägib eesti või vene keelt,
- annab oma kontaktandmed ning on nõus sellega, et uuringukeskuse töötajad võivad temaga uuringu jätkukohtumise ja järelvisiidi toimumise aja meenutamiseks ühendust võtta,
- nõustub osalema uuringu esimesel põhivisiidil, järelvisiidil (1 kuu möödudes) ja teisel põhivisiidil (6 kuu möödudes peale esimest põhivisiiti).

Uuringust väljajätmise kriteeriumid:

- osalemine mõnes teises HIV-ennetamise või narkootikumide kasutamise sekkumisuuringus,
- uuringupersonali hinnangu kohaselt (häiritus)seisund (sh alkoholi või narkojoobes olek) või kognitiivne puue, mis ei võimalda uuritaval anda teadlikku informeeritud nõusolekut või järgida uuringuvisiitide ajakava ning uuringuga seotud protseduure.

Uuringuvisiidid toimuvad uuringu toimumise ajaks renditud ruumides Tallinnas (MTÜ Convictus Eesti, Lastekodu 6, Tallinn), kus on sobivad ruumid uuritavate privaatseks intervjueerimiseks, neile planeeritud sekkumise (nõustamine) läbiviimiseks ning uuritavate kontaktandmete turvaliseks hoidmiseks. Uuringuandmete hoidmiseks on 2 rauast seifi, millele on ligipääs vaid uuringu projektijuhil, superviisoril ja vastutaval uurijal.

Enne uuringuga seotud protseduuride alustamist selgitatakse uuritavale põhjalikult uuringu sh sekkumise eesmärki, olemust, uuringuga ja sekkumises osalemisega seotud kasu ja võimalikku kahju ning uuritava konfidentsiaalsuse tagamiseks ja tema kontaktandmete turvaliseks hoidmiseks kasutatavaid meetmeid. Uuritav allkirjastab uuritava informeerimise ja teadliku nõusoleku vormi (lisatud uurimistöö taotlusele).

Selles jälgimisuuringus kogutakse uuritavatelt nende isikuandmed (ees- ja perenimi, isikukood, elukoha aadress, e-mail ja telefoninumber) ning kuni kahe lähedase (pereliige, sõber, tuttav) inimese kontaktandmed (juhuks, kui uuritavat ei ole võimalik isiklikult kätte saada näiteks seetõttu, et ta vahetab sageli oma telefoninumbrit ja elukohta või tal puudub kindel elukoht või ei kasuta ta telefoni).

Informeerimise ja teadliku nõusoleku vormi allkirjastanud uuritav annab sellega oma nõusoleku uuringus (sh sekkumises) osalemiseks, HIV/HCV-testimiseks ja tema kontaktandmete hoidmiseks ja kasutamiseks järgnevatele uuringu visiitidele kutsumiseks.

Vajaduse korral, et teha kindlaks, kas uuritav on: (1) narkootikume mitte-süstiv inimene, kontrollitakse tema nahka süstimisjälgede puudumise suhtes ja palutakse neil põhjalikult kirjeldada peamise narkootikumina kasutatava illegaalse narkootikumi ettevalmistamist tarvitamiseks; või (2) narkootikume süstiv inimene, kontrollitakse tema nahka süstimisjälgede olemasolu suhtes ja palutakse neil põhjalikult kirjeldada peamise narkootikumina süstitava illegaalse narkootikumi ettevalmistamist tarvitamiseks ja tarvitamist.

Uuringus kogutud andmete säilitamine ja käitlemine

Uuringus kogutud andmed säilitatakse peale sisestamist elektroonilisse andmebaasi. Uuritavate andmed sisestatakse kasutades umbisikulist uuritava koodi. Uuringu andmebaasi, mis sisaldab andmeid küsimustikest ja infot testitulemuste ning uuritava hinnangut sekkumise kohta ei sisestata uuritavate isikut identifitseerida võimaldavat infot. Uuringus kogutud uuritavate isikuandmed säilitatakse eraldi uuringu andmetest elektrooniliselt salasõnaga kaitstud failis. Täidetud küsimustikke, uuritava hinnangut sekkumisele ning uuringu seire vormid paberkandjal säilitatakse lukustatud kapis viis aastat (Tartu Ülikooli peremeditsiini ja rahvatervishoiu instituudi ruumides, Ravila 19, Tartu 50411).

Uuringu andmete sisestus ja käitlemine toimub ainult TÜ Peremeditsiini ja rahvatervishoiu Instituudis. Uuringus kogutud andmeid kasutatakse ainult teadusliku uurimistöö eesmärgil. Uuringus kogutud andmeid kasutatakse ka edaspidistes teadusuuringutes.

TÜ Peremeditsiini ja rahvatervishoiu Instituudis on välja töötatud dokument „Teadustöö planeerimise ja andmete töötlemise juhend lähtuvalt isikuandmete kaitse nõuetest Tartu Ülikooli peremeditsiini ja rahva Peremeditsiini ja rahvatervishoiu instituudi.

8. UURIMISMETOODIKA TÄPNE KIRJELDUS

<u>Uuringu esimene põhivisiit</u>

<u>Toimingud</u>

1. Informeeritud nõuoleku saamise protseduur (selgitatakse uuritavale põhjalikult uuringu eesmärki, olemust, uuringuga seotud kasu ja võimalikku kahju ning uuringu konfidentsiaalsuse tagamiseks kasutatavaid meetmeid. Uuritav allkirjastab informeeritud nõusoleku lehe);
2. Märgitakse üles osaleja isikuandmed (nimi/hüüdnimi, isikukood, aadress, telefoni number ja e-posti aadress) juhuks, kui temaga on vaja uuringu jooksul tekkivate oluliste küsimuste korral ühendust võtta;
3. Uuritaval palutakse anda kolme oma lähedase (pereliige) ja/või sõbra/tuttava/usaldusisiku kontaktandmed (nimi/hüüdnimi, aadress, telefoninumber ja e-posti aadress) juhuks, kui teda ei ole mingil põhjusel (vahetanud telefoni, elukohta, ei kasuta e-maili, sattunud kinnipidamisasutusse, haiguse tõttu raviasutusse, surnud) võimalik uuringuga seoses kätte saada;
4. Viiakse läbi struktureeritud intervjuu (küsimustikud süstijatele, mitte-süstijatele ja vähese süstimiskogemusega süstijatele eesti/vene keeles lisatud). Lisaks demograafilisele ja sotsiaalmajanduslikele tunnustele ja värbamise jaotusele sisaldab uuringu küsimustik valitud küsimusi narkootikumide ja alkoholi tarvitamise ja esmatarvitamise vanuse, peamise narkootikumi tarvitamise tõttu esinevate probleemide, enda narkootikumide tarvitamise, kokkupuudete kohta narkootikume süstivate inimestega (sh abi saamine ja /või teiste

abistamine esmakordse narkoosestki tegemisel), elukeskkonnaga seotud väliste normide, seksuaalse riskikäitumise ja narkootikumide kasutamisega seotud riskikäitumise, üledoosi, psühholoogilise ja füüsilise tervise, kahjude vähendamise ja narkomaania, ARV, C-hepatiidi ravivõimaluste kasutamise ja sotsiaalse toe kohta.

Intervjuu alusel määratletakse spetsiifilise sekkumise vajadus:

i. Süstivad narkomaanid saavad „Katkesta ahel“ sekkumise
ii. Mitte-süstivad narkomaanid saavad „Väldi nõela“ sekkumise

5. Küsimustele vastamiseks kulub umbes 40 minutit, intervjuu toimub vastavalt uuritava soovile kas eesti või vene keeles. Küsimustikud kolmele uuritavate rühmale (on süstijad, on vähese süstimiskogemusega, ei ole kunagi süstinud) erinevad väikeses mahus (kunagi süstinuilt küsitakse ka süstimisega seotud küsimusi) (Lisad 11.3.1 ja 11.3.2);
6. Uuritavatel viiakse läbi HIV ja HCV testi eelne nõustamine väljaõppinud töötaja poolt; kogutakse uuritavalt 15 ml veeniverd HIV- ja HCV vastaste antikehade olemasolu määramiseks. Veenivere kogub meditsiiniõe väljaõppega isik, järgides kõiki nakkuskontrolli põhimõtteid. Testimine HIV/HCV antikehade suhtes toimub SYNLAB Eesti OÜ (Veerenni 53a, 11313, Tallinn) IV põlvkonna ELISA meetodil. SYNLAB Eesti OÜ labor on akrediteeritud Eesti Akrediteerimiskeskuse poolt (akrediteerimistunnistus L159) ja järgib standardi ISO 15189 „Meditsiinilaborid. Kvaliteedi ja kompetentsi erinõuded“ nõudeid ning lähtub oma tegevuse korraldamisel Eesti Vabariigis kehtivates õigusaktidest. Skriiningtestil HIV positiivsed analüüsid verifitseeritakse Western blot analüüsil (vastavalt riiklikele eeskirjadele). Analüüside tulemused antakse uuritavale teada 7 päeva jooksul peale testi tegemist;
1. Uuringukeskuse superviisor lepib uuringus osalejaga kokku täpse järgmise visiidi aja.

Uuringunõustamine (sekkumised)

Uuringus testitav sekkumine “Väldi nõela” koosneb kahest individuaalsest sekkumise (nõustamise) sessioonist, mille viib läbi sekkuja. Esimese kestus on ~ 60 minutit ja teise (mis toimub 4 nädala möödudes) kestus ca 20 minutit. Selles sekkumises osalevad kas narkootikume mitte kunagi süstinud või vähese süstimiskogemusega, kuid viimase kahe kuu jooksul neid muul viisil, kui süstides tarvitavad uuritavad.

Taotluse lisadena on esitatud süstijatele suunatud (Lisa 11.4.1.3), mitte kunagi süstinutele suunatud (Lisa 11.4.1.1) ning vähese süstimiskogemusega isikule suunatud (Lisa 11.4.1.2) sekkumisjuhised.

Uuritava nõusolekul sekkumise sessioonid lindistatakse sekkumise rakendamise täpsuse (*ingl* intervention fidelity) uurimiseks. Lisaks palutakse uuritavalt täita tagasiside vorm andmaks hinnang sekkumisele.

Uuringu teine põhivisiit

Teisel põhivisiidil (6 kuud peale esimese põhivisiidi toimumist) osalevad kõik uuritavad.

Toimingud

1. Viiakse läbi struktureeritud intervjuu, mis sisaldab valitud küsimusi süstimisega alustamise ja teistel alustamise assisteerimise, uuritava seksuaalse riskikäitumise ja narkootikumide kasutamisega seotud riskikäitumise, kriminaalse tausta ja narkomaania ravivõimaluste kasutamise kohta. Küsimustele vastamiseks kulub umbes 45 minutit, intervjuu toimub vastavalt uuritava soovile kas eesti või vene keeles;
2. Kui uuritava HIV-ja/või HCV testi tulemused oli eelmisel visiidil negatiivne, palutakse tal anda uus vereanalüüs HIV/HCV-vastaste antikehade määramiseks teisel põhivisiidil (6 kuud peale esimese põhivisiidi toimumist);
3. Analüüside tulemused antakse uuritavale teada testijärgse nõustamise raames hiljemalt 1 nädala pärast tema testimist HIVi ja C-hepatiidi suhtes. Positiivse testitulemuse korral aidatakse suunata uuritav sobivale teenusele LTKH nakkuskeskuses ARV raviga tegeleva infektsionistiga ja / või antakse talle infot ja/või abistatakse teda võtma ühendust C-hepatiidi raviga tegeleva gastroenteroloogiga.

9. UURIMISTÖÖ EETILISTE ASPEKTIDE KIRJELDUS

Tegemist on eksperimentaalse uuringuga, kus uuritavad kuuluvad häbimärgistatud (*ingl* stigmatized) rahvastiku rühma oma narkootikumide tarvitamise tõttu. On väga oluline tagada uuritavate autonoomsus, uuringus osalemise vabatahtlikkus ning väärikas ja lugupidav kohtlemine.
Oleme põhjalikult läbi analüüsinud uuringus osalemisega seotud võimalikud riskid ning rakendame nende miniseerimiseks arvestavaid ning tunnustatud meetmeid.

Uuringuga seotud võimalik kahju
(1) isiku privaatsuse kadu;
(2) kogutud andmete konfidentsiaalsuse kadu;
(3) füüsiline ebamugavus vereproovi võtmisel;
(4) psühholoogiline ebamugavus seoses narkootikumide kasutamist ja seksuaalelu puudutavatele küsimustele vastamisega;
(6) sotsiaalsed kahjud seoses uuringus osalemisega (nt häbimärgistamine, diskrimineerimine narkootikuminde kasutaja staatuse või seksuaalkäitumise tõttu).

Uuringupersonal on nimetatud sihtrühmaga töötamises väga kogenud ja osalenud varem narkootikumide süstijate hulgas eksperimentaalsete sekkumisuuringute “Katkesta ahel” ja “Väldi nõela” läbiviimisel. Uuringus töötavad inimesed (superviisor, projektijuht, intervjueerijad, sekkujad) on saanud ka sihtrühmaga suhtlemiseks vajaliku motiveeriva intervjueerimise koolituse, mis tagab uuritavate väärika, nende autonoomsust arvestava ja lugupidava suhtumise. Head tööõhkkonda aitavad tagada regulaarsed supervisioonid sekkujatele ja kogu uuringumeeskonna regulaarsed kohtumised uuringuga seotud küsimuste arutamiseks ja nende lahendamiseks.
Uuring privaatsetes ruumides on olemas vajalikud tingimused intervjueerimiseks, sekkumise läbiviimiseks ning HIVi ja C-hepatiidi suhtes testimiseks.

Uuringus kogutud andmed säilitatakse andmebaasi uuritavate kodeeritud uuringutulemustega (vastused intervjuu küsimustele, analüüside tulemused, hinnang sekkumisele) elektrooniliselt salasõnaga kaitstud failis. Täidetud küsimustikud ning uuringu monitooringu vormid paberkandjal säilitatakse lukustatud kapis viis aastat. Uuringu andmed elektroonilises formaadis säilitatakse tähtajatult.

Võimaliku psühholoogilise ebamugavuse vähendamiseks on uuringupersonal saanud eelnevalt uuringu läbiviimise koolituse ning kõigile uuritavatele antakse uuringu koordinaatori ja superviisori kontaktandmed. Uuritavad võivad pöörduda koordinaatori poole mistahes uuringut ja/või narkootikumide kasutamist puudutavate küsimustega.

Uuritavatele jagatakse ka infovoldik narkootikumide tarbimise vähendamise ja HIV/AIDSi-alase nõustamis- ja ennetustööga tegelevate keskuste kontaktandmetega Tallinnas. Uuritavatele antakse mõlema sekkumise raames infoleht erinevate narkootikumide üledoosi ennetamise kohta. Lisaks antakse neile MTÜ Convictuse kontaktandmed ning nende soovil lepitakse nendega kokku naloksooni koolituse aeg (toimub üks kord nädalas MTÜ Convictus Eesti ruumides).

Sotsiaalsete kahjude minimeerimiseks rakendatakse kõik meetmed kaitsmaks uuritava isikuandmeid ning uuringu käigus kogutud teavet. Psühholoogilise distressi korral on uuritavale alati kättesaadav spetsiaalse väljaõppega uuringupersonal, et arutada tekkinud probleeme ning leida lahendusi sh suunata MTÜ Convictus psühholoogi tasuta vastuvõtule.

Uuritavatele pakutakse positiivse HIVi või C-hepatiiditesti korral nõustamist. Uuringu meditsiiniõde arutab koos uuritavaga erinevaid ravile pöördumise võimalusi ning vajadusel lepib kokku arstivisiidi nakkuskeskusesse.

Uuringust saadav kasu
Uuringus kogutav informatsioon annab võimaluse hinnata narkootikumidega süstimist ennetava ja/või vähendava sekkumise teostatavust ja selle tõhusust ning täiendavalt monitoorida HIVi ja C-hepatiidi levikut sellest epideemiast haaratud rahvastikurühmas. Uuringu tulemusena saadakse väärtuslikku

infot sihtrühmale sobivate ennetusmeetmete ning terviseteenuste ja kahjude vähendamise teenuste loomiseks.

Kasu uuringus osalejale
Osalus uuringus võimaldab narkootikume süstivatel ja mitte süstivate uuritavate suunamist sobivate teenuste juurde ja nende kohta informatsiooni jagamist (suunamine võõrutus-või asendusravile, kaksikdiagnoosiga inimeste raviga tegelevasse polikliinikusse ja keskusesse, HIV meditsiinilisele ravile, sotsiaalsele rehabilitatsioonile). Lisaks jagatakse uuritavatele infomaterjale ja tasuta kondoome.

10. TEAVE SAMA PROJEKTI VARASEMATEST VÕI SAMAAEGSETEST HINDAMISTEST VÕI HEAKSKIITMISTEST MUJAL.

Uuritavate poolt juhitud kaasamine on valideeritud uuringusse kaasamise metoodikaga, mida on korduvalt ja edukalt rakendatud nii rahvusvaheliselt kui ka Eestis. Uuringus kasutatavad sekkumised on valideeritud ja rakendatud ka teistes riikides sh Eestis. Analoogilise uuringu läbiviimise taotlus on esitamisel ka USA poolse partneri asutus – Mount Sinai Beth Israel Medial center – eetikakomiteele.

11. LISAD:

11.1 Vastutava uurija ja kaasuurijate CV
Anneli Uusküla, Ave Talu, Greete Org

11.2 Uuritava informeerimise ja teadliku nõusoleku vormid (eesti ja vene keeles)

11.3 Uuringuküsimustikud (eesti ja vene keeles)
11.3.1 Küsimustik kunagi süstinuile
11.3.2 Küsimutik mitte kunagi süstinuile

11.4 Nõustamisjuhised (eesti ja vene keeles)
11.4.1 Nõustamisjuhis esimene sessioon
11.4.1.1 Juhis kunagi süstinuile
11.4.1.2 Juhis mitte kunagi süstinuile
11.4.1.3 Juhis süstijale
11.4.2 Nõustamisjuhis teine sessioon

11.5 Infovoldikute näidised

11.6 Uuringus osalemise võimaluse tutvustus narkomaania ravi keskustes jm.

Lisa 11.1

Anneli Uusküla, CV

I. ISIKUANDMED

1. Nimi	ANNELI UUSKÜLA
2. Sünniaeg	11.04.1967
3. Kodakondsus	Eesti
4. Aadress, telefon, e-post	Veski 26-3, Tartu 50409; +372 518 3552; anneli.uuskula@ut.ee
5. Haridus	SUNY, School of Public Health at Albany, NY, USA, teadusmagistrikraad (MS Epidemiology), 2003<br>Tartu Ülikool, meditsiinidoktor, 2001<br>Tartu Ülikool, eriala residentuur (dermatovenerolooogia), 1997<br>Tartu Ülikool, üldinternatuur, 1993<br>Tartu Ülikool, arstiteaduskond, 1991
6. Keelteoskus	Eesti, inglise, vene
7. Teenistuskäik	2007. a alates, TÜ tervishoiu instituut, epidemioloogiaprofessor<br>2007–2009, TÜ tervishoiu instituut, juhataja<br>2000. a alates, TÜ nahahaiguste kliinik, arst-õppejõud<br>2004–2007, TÜ tervishoiu instituut, vanemteadur<br>2004–2007, TÜ nahahaiguste kliinik, vanemassistent<br>2002–2003, TÜ tervishoiu instituut, erakorraline teadur<br>1994–1995, TÜ nahahaiguste kliinik, vanemlaborant

II. TEADUSTÖÖ JA ARENDUSTEGEVUS

8. Peamised uurimisvaldkonnad

Olulisteks uuringuvaldkondadeks on (i) nakkushaiguste (sugulisel teel levivad infektsioonid, HIV) ja riskeeriva käitumise epidemioloogilised uuringud; (ii) tervist edendavate ja kahjusid vähendavate sekkumiste hindamine. Tervist edendavaid sekkumisi (antiretroviirusravi (ART) järgimise toetamine, juhtumikorralduse metoodikad) on hinnatud indiviidi tasemel, kasutades juhuslikustatud kontrollitud katsemetoodikat; (iii) teaduskirjanduse süstemaatiline analüüs ning sünteesimetoodikate (kvalitatiivsete, kvantitatiivsete) omandamine.

9. Teaduspublikatsioonid

H-indeks: 16 (ISI Web of Knowledge, 27.05.2017).

10. Publikatsioonid rahvusvahelistes eelretsenseeritavates teadusajakirjades (2017-2018)

1: Uusküla A, Barnes DM, Raag M, Talu A, Tross S, Des Jarlais DC. Frequency and factors associated with providing injection initiation assistance in Tallinn, Estonia. Drug Alcohol Depend. 2018 May 8;188:64-70.

2: Uusküla A, Raag M, Vorobjov S, Jarlais DD. Another frontier for harm reduction: contraceptive needs of females who inject drugs in Estonia, a cross-sectional study. Harm Reduct J. 2018 Mar 5;15(1):10.

3: Tavitian-Exley I, Maheu-Giroux M, Platt L, Heimer R, Uusküla A, Levina O, Vickerman P, Boily MC. Differences in risk behaviours and HIV status between primary amphetamines and opioid injectors in Estonia and Russia. Int J Drug Policy. 2018 Mar;53:96-105.

4: Svedbom A, Borgström F, Hernlund E, Ström O, Alekna V, Bianchi ML, Clark P, Curiel MD, Dimai HP, Jürisson M, Uusküla A, Lember M, Kallikorm R, Lesnyak O, McCloskey E, Ershova O, Sanders KM, Silverman S, Tamulaitiene M, Thomas T, Tosteson ANA, Jönsson B, Kanis JA. Quality of life after hip, vertebral, and distal forearm fragility fractures measured using the EQ-5D-3L, EQ-VAS, and time-trade-off: results from the ICUROS. Qual Life Res. 2018 Mar;27(3):707-716.

5: Svedbom A, Borgstöm F, Hernlund E, Ström O, Alekna V, Bianchi ML, Clark P, Curiel MD, Dimai HP, Jürisson M, Kallikorm R, Lember M, Lesnyak O, McCloskey E, Sanders KM, Silverman S,

Solodovnikov A, Tamulaitiene M, Thomas T, Toroptsova N, Uusküla A, Tosteson ANA, Jönsson B, Kanis JA. Quality of life for up to 18 months after low-energy hip, vertebral, and distal forearm fractures-results from the ICUROS. Osteoporos Int. 2018 Mar;29(3):557-566.
6: Wu J, Crawford FW, Raag M, Heimer R, Uusküla A. Using data from respondent-driven sampling studies to estimate the number of people who inject drugs: Application to the Kohtla-Järve region of Estonia. PLoS One. 2017 Nov 2;12(11):e0185711. doi: 10.1371/journal.pone.0185711.
7: Tisler-Sala A, Ojavee SE, Uusküla A. Treatment of chlamydia and gonorrhoea, compliance with treatment guidelines and factors associatedwith non-compliant prescribing: findings form a cross-sectional study. Sex Transm Infect. 2018 Jun;94(4):298-303.
8: McNulty C, Ricketts EJ, Fredlund H, Uusküla A, Town K, Rugman C, Tisler-Sala A, Mani A, Dunais B, Folkard K, Allison R, Touboul P. Qualitative interviews with healthcare staff in four European countries to inform adaptation of an intervention to increase chlamydia testing. BMJ Open. 2017 Sep 25;7(9):e017528.
9: Werb D, Bluthenthal RN, Kolla G, Strike C, Kral AH, Uusküla A, Des Jarlais D. Preventing Injection Drug use Initiation: State of the Evidence and Opportunities for the Future. J Urban Health. 2018 Feb;95(1):91-98.
10: Võrno T, Lutsar K, Uusküla A, Padrik L, Raud T, Reile R, Nahkur O, Kiivet RA. -effectiveness of HPV vaccination in the context of high cervical cancer incidence and low screening coverage. Vaccine. 2017 Nov 1;35(46):6329-6335.
11: Jürisson M, Raag M, Kallikorm R, Lember M, Uusküla A. The impact of comorbidities on hip fracture mortality: a retrospective population-based cohort study. Arch Osteoporos. 2017 Aug 28;12(1):76.
12: Jürisson M, Raag M, Kallikorm R, Lember M, Uusküla A. Erratum to: The impact of hip fracture on mortality in Estonia: a retrospective population-based cohort study. BMC Musculoskelet Disord. 2017 Aug 24;18(1):366.
13: Uusküla A, Laisaar KT, Raag M, Lemsalu L, Lõhmus L, Rüütel K, Amico KR; HIV-BRIDGE Study Group. Effects of Counselling on Adherence to Antiretroviral Treatment Among People with HIV in Estonia: A Randomized Controlled Trial. AIDS Behav. 2018 Jan;22(1):224-233.
14: Tavitian-Exley I, Boily MC, Heimer R, Uusküla A, Levina O, Maheu-Giroux M. Polydrug Use and Heterogeneity in HIV Risk Among People Who Inject Drugs in Estonia and Russia: A Latent Class Analysis. AIDS Behav. 2018 Apr;22(4):1329-1340.
15: Chan PY, Joseph MA, Des Jarlais DC, Uusküla A. Perceived effectiveness of antiretroviral therapy, self-rated health and treatment adherence among HIV-positive people who inject drugs in Estonia. Int J STD AIDS. 2018 Jan;29(1):13-22.
16: Jürisson M, Raag M, Kallikorm R, Lember M, Uusküla A. The impact of hip fracture on mortality in Estonia: a retrospective population-based cohort study. BMC Musculoskelet Disord. 2017 Jun 5;18(1):243. doi: 10.1186/s12891-017-1606-1. Erratum in: BMC Musculoskelet Disord. 2017 Aug 24;18(1):366.
17: Wiessing L, Ferri M, Běláčková V, Carrieri P, Friedman SR, Folch C, Dolan K, Galvin B, Vickerman P, Lazarus JV, Mravčík V, Kretzschmar M, Sypsa V, Sarasa-Renedo A, Uusküla A, Paraskevis D, Mendão L, Rossi D, van Gelder N, Mitcheson L, Paoli L, Gomez CD, Milhet M, Dascalu N, Knight J, Hay G, Kalamara E, Simon R; EUBEST working group, Comiskey C, Rossi C, Griffiths P. Monitoring quality and coverage of harm reduction services for people who use drugs: a consensus study. Harm Reduct J. 2017 Apr 22;14(1):19.
18: Heimer R, Usacheva N, Barbour R, Niccolai LM, Uusküla A, Levina OS. Engagement in HIV care and its correlates among people who inject drugs in St Petersburg, Russian Federation and Kohtla-Järve, Estonia. Addiction. 2017 Aug;112(8):1421-1431.
19: Uusküla A, Raag M, Marsh K, Talu A, Vorobjov S, Des Jarlais D. HIV prevalence and gender differences among new injection-drug-users in Tallinn, Estonia: A persisting problem in a stable high prevalence epidemic. PLoS One. 2017 Feb 2;12(2):e0170956.
20: Johannson A, Vorobjov S, Heimer R, Dovidio JF, Uusküla A. The Role of Internalized Stigma in the Disclosure of Injecting Drug Use Among People Who Inject Drugs and Self-Report as HIV-Positive in Kohtla-Järve, Estonia. AIDS Behav. 2017 Apr;21(4):1034-1043.
21: Lemsalu L, Rüütel K, Laisaar KT, Lõhmus L, Raidvee A, Uusküla A; HIV-BRIDGE Study Group. Suicidal Behavior Among People Living with HIV (PLHIV) in Medical Care in Estonia and Factors Associated with Receiving Psychological Treatment. AIDS Behav. 2017 Jun;21(6):1709-1716.
22: Jõgeda EL, Huik K, Pauskar M, Kallas E, Karki T, Des Jarlais D, Uusküla A, Lutsar I, Avi R. Prevalence and genotypes of GBV-C and its associations with HIV infection among persons who inject drugs in Eastern Europe. J Med Virol. 2017 Apr;89(4):632-638. 2012: a cross-national survey. Eur J Public Health. 2016 Jun;26(3):382-8.

11. Taotlusperioodi uurimistoetused ja lepingud (uuritavate arvud)

Pealkiri	Periood	Uuritavate arv	Rahastaja
Combined prevention to reduce initiation into injecting drug use	2015-2020	375	NIH/USA Grant DP1DA039542
Health research in the continuum of the evidence based practice in Estonia	2015-2020	Institutsionaane uurimisgrant	Haridus- ja Teadusministeerium

12. Läbitud eetikaalased koolitused (viimased 5 aastat)
    2011, training in human subjects protection [Sean Philpott/Union Graduate College]
    2013, CITI Program's Human Subjects Research (HSR) (https://www.citiprogram.org/index.cfm?pageID=88) kursused

Anneli Uusküla
22. mai 2**018.a.**

Ave Talu, CV

ISIKUANDMED
Nimi: Ave Talu
Telefon: +372 5227267
e-mail: ave.talu@gmail.com; ave.talu@ut.ee

HARIDUS
2004–2008 Tartu Ülikool, teadusmagistri kraad (*magister scientarium*) rahvatervise erialal (MScPH), õppekavajärgne spetsialiseerumine: epidemioloogia ja biostatistika;
1991–1995 Tallinna Pedagoogikaülikool, eelkooli pedagoogika ja psühholoogia õpetaja, BA;
1992–1996 Tallinna Pedagoogikaülikool, sotsiaaltöö, BA.

TÖÖKOGEMUS
2013 – .... sõltumatu ekspert (rahvatervishoiu valdkonna uuringud, hindamine ja seire).

Käimasolevad projektid:

1) Tartu Ülikooli peremeditsiini ja rahvatervishoiu instituudi uuring "Kombineeritud sekkumine "Väldi nõela" süstimisega alustamise vähendamiseks " (2017 – ....);

Lõpetatud projektid:

2) Tartu Ülikooli peremeditsiini ja rahvatervishoiu instituudi uuring "Kombineeritud sekkumine vähendamaks süstimisega alustamist"(2016 – 2017);
3) Tartu Ülikooli sotsiaalteaduslike rakendusuuringute keskus, uuring "Narkosõltlastest õigusrikkujate sõltuvusravi ja rehabilitatsioon Eestis" (2015 – 2016);
4) Tartu Ülikooli haridusuuringute ja õppekavaarenduse keskus, uuring "Terviseedenduse tulemuslikkus Eesti koolides" (TerVEkool) (2014 – 2015);
5) USA Kaitseministeeriumi HIV/AIDSi ennetamise projekt Eesti Kaitseväes (Department of Defence HIV/AIDS Prevention Program) (2014 – 2016);
6) Tartu Ülikooli tervishoiu instituudi uuring süstivatele narkomaanidele suunatud kombineeritud HIVi nakatumist vähendavate sekkumiste teostatavusest ja sobivusest (2013 – 2014).

2010 – .... ResAd (Tšehhi Vabariik), Euroopa Liidu narkoalane tegevusprogramm Kesk-Aasias (Central Asia Drug Action Programme, CADAP 5, CADAP 6), narkoseire ja uuringute ekspert,

CADAP 6 töögrupi juht Tadžikistanis;

2002 – 2013 Tervise Arengu Instituut, Eesti Uimastiseire Keskus / EMCDDA Eesti Reitox narkoteabe keskus, juhataja;
2001 – 2002 Eksperimentaalse ja Kliinilise Meditsiini Instituut (2003. aastast Tervise Arengu Instituut), Eesti Uimastiseire Keskus / EMCDDA Eesti Reitox narkoteabe keskus, spetsialist;
1998 – 2001 Eesti Uimastipreventsiooni Sihtasutus, projektijuht;
1998 – 1998 Justiitsministeerium, kriminaalpreventsiooni talitus, peaspetsialist;
1998 Kristiine Linnaosa Valitsus, lastekaitse vaneminspektor;
1996 – 1998 Sotsiaalministeerium, mittekoosseisuline peaspetsialist.

KEELTEOSKUS
eesti keel – emakeel;
inglise keel – tööalane suhtluskeel;
vene keel – tööalane suhtluskeel.

ARVUTIOSKUS
Microsift Office™, Epiinfo, STATA.

KOOLITUS
2006 Fogarty stipendium HIV/AIDSi ja narkouuringute alal, USA (Fogarty Fellowships on HIV/AIDS and drug research, USA, New York, National Development and Research Institute, Centre for Drug Use and HIV Research; Fogarty International Centre Grant D43TW0000233);
2002 Pompidou Group'i narkouuringute stipendium (Pompidou Group Fellowship under the Pompidou Group Fellowship Scheme for Studies and Research in Drug Abuse

LÄBITUD EETIKAALASED KOOLITUSED
2013, CITI Program's Human Subjects Research (HSR) (https://www.citiprogram.org/index.cfm?pageID=88) kursused

Greete Org, CV

Sünniaeg: 09.02.1988, Tallinn
Aadress: Sõstra 4-35, 10616 Tallinn
Telefon: +372 58 25 80 65 (mobiil)
E-post: greete.org@mail.ee

Hariduskäik:
2016 – … Tallinna Ülikool. Ühiskonnateaduste Instituut. Sotsiaaltöö.
Õppevorm: tsükkelõpe. Eeldatav lõpetamise aeg 2019
2013 – 2015 Tallinna Täiskasvanute Gümnaasium, 11-12 klass
1995 – 2007 Tallinna Arte Gümnaasium, 1-11 klass

Täiendkoolitus:
august 2011 - HIV/AIDSi ja narkomaania ennetamise valdkonnas tegutsevate organisatsioonide ja nende koostööpartrnerite suvekool. Tervise Arengu Instituut, maht 13 tundi
märts 2012 - Tuberkuloos kui sotsiaalse taustaga nakkushaigus, Tervise Arengu Instituut, maht 4 tundi
juuni 2012 - HIV, hepatiit, tuberkuloos, seksuaalsel teel levivad haigused, Tervise Arengu Instituut, maht 7 tundi
oktoober 2012 HIV ja naine, Tervise Arengu Instituut, maht 6 tundi
august 2014 - HIV/AIDSi ja narkomaania ennetamise valdkonnas tegutsevate organisatsioonide ja nende koostööpartrnerite suvekool. Tervise Arengu Instituut, maht 12 tundi
oktoober 2014 - HIV/AIDSi ja narkomaania ennetamise valdkonnas tegutsevate organisatsioonide ja

nende koostööpartrnerite suvekool. Tervise Arengu Instituut, maht 12 tundi
oktoober 2014 - Toimetulek agressiivse käitumisega. Verge Eesti, maht 14 tundi
aprill 2015 - Uute töötajate juhtumiarutelu kohtumiste läbiviijate koolitus. Tervise Arengu Instituut, maht 6 tundi
aprill 2016 - Koolitusel esinemine ja õppimise toetamine. Tervise Arengu Instituut, maht 16 tundi
august 2016 - HIV/AIDSi ja narkomaania ennetamise valdkonnas tegutsevate organisatsioonide ja nende koostööpartrnerite suvekool. Tervise Arengu Instituut, maht 13 tundi
november 2016 - Läbipõlemise ennetamine. MTÜ Evrika, maht 20 tundi
november 2016 - Uimastid ja sõltuvus. Tervise Arengu Instituut, maht 8 tundi
detsember 2016 - Uimastid ja sõltuvus. Tervise Arengu Instituut, maht 8 tundi
detsember 2016 - Kuidas võita sõpru ja mõjutada inimesi töötades HIV ja narkomaania valdkonnas. Tervise Arengu Instituut, maht 6 tundi
märts 2017 - Infektsioonid ja muud tööga seotud ohud. Tervise Arengu Instituut, maht 5 tundi
juuli 2017 - Väldi nõela, sekkumine. Tartu Ülikool, Mount Sinai, maht 20 tundi
august 2017- Motiveeriv interjueerimine. Algtase. EMITA Kool, maht 20 tundi Motiveeriv interjueerimine. Algtase. EMITA Kool, maht 20 tundi
november 2017 - HIV ja HCV – infektsioonidest, diagnoosimisest ja nõustamisest. SYNLAB, maht 3 tundi
aprill 2018 - International school. Treatment and rehab for addicts. Workshop topics: alcoholism in family: diagnosis, consequences, correvtive, and preventive work, Program 12 steps- treatment and rehabilitation. Training company: Open Society Institute- NY/Budapest Regional Alcohol & Drug Program Fond, Stefan Batorego. Poland, Warsaw
mai 2018 - IFFERENT WAYS TO OVERCOME CHALLENGES – OUR RESPONSES. 2^{d} International workshop Joint Action on HIV and Co-Infection Prevention and Harm Reduction HA-REACT. Utrecht, Netherlands

Lisa 11. Informeeritud nõusoleku vormid.

„Kombineeritud sekkumine süstimisega alustamise vähendamiseks"

UURITAVA INFORMEERIMISE JA TEADLIKU NÕUSOLEKU LEHT

Kutsume Teid osalema teadusuuringus, millega püüame leida uusi ja paremaid meetodeid süstitavate narkootikumide kasutamise ja HIVi (inimese immuunpuudulikkuse viirus – *human immunodeficiency virus* –, mis põhjustab AIDSi) levimise aeglustamiseks narkootikume kasutavate isikute seas.

Uuringud on näidanud, et osa narkootikumide kasutajaid alustab siiski ka narkootikumide süstimist. Narkootikume süstivatel isikutel on suurem tõenäosus HIVi nakatuda kui teistel. Paljusid meist ei ole kunagi HIVi suhtes testitud.

Samuti on uuringutest selgunud, et narkootikumide kasutajad satuvad vahel olukordadesse, kus soovitatakse või toetatakse esimese narkootikumisüsti tegemist. Samuti võivad inimesed, kes ise narkootikume ei süsti, sattuda seltskonda, kus räägitakse tunnustavalt narkootikumide süstimisest, rõhutades selle subjektiivseid tugevaid külgi, või süstitakse teiste (mittesüstijate) inimeste juuresolekul. Selles uuringus kasutatava nõustamise eesmärk ongi selliseid olukordi arutada ja suunata narkootikumide kasutajaid neist hoiduma.

Ka on erinevad uuringud on näidanud, et vahel süstivad narkomaanid räägivad tunnustavalt narkootikumide süstimisest, rõhutades selle kasutamise viisi subjektiivseid tugevusi või süstivad inimeste juuresolekul, kes ise ei kasuta narkootikume või siis ise ei süsti. Lisaks vahel süstijad abistavad teisi esimese narkootikumide süsti tegemisel. Sekkumise eesmärgiks ongi selliseid olukordi arutada ja suunata narkootikume süstijaid neist hoiduma.

Miks seda uuringut tehakse?

See uuring korraldatakse, et saada teavet narkootikumide süstimisega seotud narkomaania kohta. Seejuures keskendutakse süstimise alustamisega või sellega mittealustamisega seotud teguritele. Uuringu käigus läbi viidava sekkumise eesmärk on ennetada narkootikumide süstimise alustamist. Arutatakse ka, millised riskid kaasnevad esimese narkosüsti tegemisega. Lisaks antakse sekkumise käigus teavet ohutu süstimise ja üledooside ennetamise kohta.

Uuringusse kaasatakse kokku 300 isikut, kes on narkootikume kasutanud viimasel kahel kuul, kuid ei ole neid tarvitanud süstides ning kes elavad Tallinnas või Harjumaal. Kutsume Teidki uuringusse.

Enne kui otsustate, kas uuringus osaleda, peaksite teadma, millised on võimalikud uuringuga seotud riskid ning kasutegurid. Siinsel teadliku nõusoleku vormil on antud Teile teavet selle kohta, mis Teiega uuringu ajal toimub, ning selgitatud põhjalikult kõike uuringuga seonduvat. Selles dokumendis võib olla kasutatud sõnu või termineid, mida Te ei tea. Enne nõusolekuvormi allkirjastamist küsige palun selgitust kõige kohta, mis ei ole Teile selge. Kui Te kinnitate, et saate uuringust aru, ning olete otsustanud selles osaleda, palutakse Teil nõusolekuvorm läbi lugeda ning allkirjastada see.

Esmalt on väga oluline teada järgmist:

- Uuringus osalemine on vabatahtlik.
- Te võite igal ajal otsustada, et lõpetate uuringus osalemise.
- Mõned inimesed ei tarvitse uuringusse sobida teabe tõttu, mis on ilmnenud uuringusse sobivuse selgitamise käigus.

Mis Teiega juhtub, kui otsustate selles uuringus osaleda?

Uuringusse sobivuse selgitamine

Teile esitatakse küsimusi narkootikumide tarvitamise kohta. Me küsime, kas Te elate Tallinna piirkonnas. See teave on vajalik, et otsustada, kas Te sobite uuringus osalema. Uuringusse mittesobimise korral ei pea keskuse personal Teile selle põhjusi selgitama.

„Kombineeritud sekkumine süstimisega alustamise vähendamiseks"

Uuringusse kaasamine
Kui Te nõustute uuringus osalema, kaasatakse Teid täna uuringusse ning Teil palutakse järgmisele visiidile 6 kuu möödudes. Esimesel järelvisiidil kohtute nõustajaga ning teine järelvisiit koosneb küsitlusest ja uue vereproovi võtmisest HIV ja HCV (C-hepatiidi viirus) testimiseks.

Esimene visiit (toimub täna ja kestab umbes 1,5 tundi)
Esiteks, Teilt küsitakse Teie elukoha ning teiste kontaktandmete kohta, et edaspidi Teiega ühendust võtta. Seda infot kasutatakse selleks, et tuletada Teile meelde järelvisiidi aega 1 ja 6 kuu möödudes tänasest visiidist. Me küsime Teilt ka Teile usaldusväärse inimese kontaktandmeid (pereliige või sõber), kellega võime ühendust võtta, kui meil on vaja Teid seoses järelvisiidiga kätte saada. Palume, et annaksite meile teada, millist suhtlusviisi eelistate, kui uuringupersonal soovib teiega ühendust võtta. Uuringukeskuse personal selgitab, mis meetmeid kasutades kaitstakse Teie konfidentsiaalsust. Selle vormi lõpus küsitakse Teilt, kas Te lubate endaga ühendust võtta.

Intervjuu küsitlejaga kestab umbes 40 minutit. Intervjueerija küsib Teie varasemate haiguste, seksuaalelu ja narkootikumide kasutamise kohta. Seoses riskeeriva käitumisega esitatakse küsimusi narkootikumide süstimise, narkomaania ja HIVi ravi ning üledooside kohta. Samuti uuritakse Teie arvamust erinevate teemade kohta. Mõned küsimused on isiklikud. Kui Te ei soovi, siis ei pea Te ühelegi neist vastama.
Teiseks, Teil palutakse osaleda sekkumises (nõustamisel), mille põhieesmärk on vähendada võimalust, et te toetate kellegi teise või alustate ise narkootikumide süstimist. Teile antakse nõu, kuidas süstimise alustamist toetavaid olukordi vältida. Lisaks jagatakse teadmisi ohutu süstimise ja üledooside ennetamise kohta. Sekkumine kestab alla 40 minuti. Teie loal nõustamine lindistatakse ja lindistused säilitatakse uuringu lõpuni.
Kolmandaks, Teil palutakse anda 10 ml verd. Vereanalüüsi põhjal testitakse HIV- ja HCV-vastaseid antikehi. Testitulemuste teatamine ning testijärgse nõustamine toimub esimese järelvisiidi ajal.
Neljandaks, Teile tutvustatakse täna soovi korral ennetus- ja tervishoiuteenuste pakkujaid Tallinnas. Näiteks kui Teie HIV-test on positiivne, saadame Teid edasi Lääne-Tallinna Keskhaigla nakkuskeskusesse. Lisaks räägitakse Teile, kuidas kaitsta ennast ja oma seksuaalpartnereid HIVi ja sugulisel teel levivate haiguste eest. Soovi korral aitavad uuringu töötajad Teil kokku leppida ka aja üledooside ennetamise koolitusel osalemiseks.

Veel lepitakse Teiega kokku järgmise visiidi aeg.

Uuritavail, kes ei ole narkootikume süstinud või kellel on väga vähene süstimise kogemus palutakse pöörduda nõustamise teisele sessioonile 1 kuu möödudes, et kohtuda nõustajaga ning arutada, kas Teil on möödunud kuul olnud olukordi, mis võinuksid süstimise alustamist toetada, Samuti uuritakse, millise kogemuse olete seejuures saanud ning kuidas on nõustamisel õpitust olnud kasu.
Teine järelvisiit 6. kuul
Teil palutakse pöörduda järelvisiidile 6 kuu möödudes. Selle käigus toimub uus küsitlus, kus uuritakse muu hulgas, milline on Teie kogemus seoses olukordadega, mis võinuksid süstimise alustamist toetada. Lisaks võetakse Teilt 10 ml verd HIV- ja HCV-vastaste antikehade määramiseks.

Juhul kui Te ei tule järelvisiidile, püüavad uuringukeskuse töötajad, lähtudes eelnevast kokkuleppest Teiega, kätte saada Teid või Teile usaldusväärset isikut, et leppida kokku uus visiidiaeg.
Teie poolt nimetatud usaldusväärse isiku poole pöördume viisi, mille lepime kokku täna. Kontaktivõtmise eesmärgiks on paluda sellel usaldusväärsel isikul Teile meenutada uuringus osalemist või uuendata Teie kontaktandmed (nt telefoninumber).

Millised on selles uuringus osalemisega kaasnevad võimalikud riskid?
Mõni inimene tunneb ennast ebamugavalt, kui talle esitatakse isiklikke küsimusi. Pidage meeles, et Te ei pea ühelegi küsimusele vastama, kui Te seda ei soovi. Me tagame Teie antud info

konfidentsiaalsuse.

Vere võtmisega seotud riskid võivad olla minestamine, valu ja/või verevalumi tekkimine nõelatorke kohas. Vereproovi võtab selleks koolitatud ja kvalifitseeritud medõde. Kui analüüside tulemused näitavad, et Teil on HIV või HCV, võib see teadmine põhjustada Teis ängistust.

Meetmed, mida uuringu töötajad kasutavad Teie isikuandmete privaatsuse ja konfidentsiaalsuse kaitsmiseks, on kirjeldatud allpool.

Kuidas kaitstakse Teie privaatsust? Mida tehakse infoga, mille olete meile usaldanud?
Privaatsuse kaitsmise eesmärgil antakse Teile uuringus osalemiseks uuringukood. Uuringu küsimustik, uuringuanalüüside saatekiri laborile ja sekkumisega seotud materjalid (sh lindistus) ei sisalda Teie nime. Teilt kogutud info sisestatakse salasõnaga kaitstud elektroonilisse andmebaasi, koos andmetega ei säilitata Teie isikut identifitseerida võimaldavad infot. Kogutud andmeid hoitakse lukustatud kapis ja neile on juurdepääs ainult uuringuga seotud töötajatel.
Uuringus kogutud andmeid säilitatakse tähtajatult ning kasutatakse ka edaspidistes teadusuuringutes ning seostamiseks teiste andmeallikatega.
Teid ei ole võimalik tuvastada nime järgi üheski selle uuringu aruandes ega publikatsioonis. Teie uuringuandmeid võivad näha uuringu sponsori (USA Riiklik Terviseinstituut) esindajad.

Uuringu on kooskõlastanud Tartu Ülikooli eetikakomitee ja uuringumeeskond on saanud põhjaliku väljaõppe.
Uuringus kogutud andmed säilitatakse ning kasutatakse vaid teaduslike eesmärkidel ka edaspidises teadustöös.

Milline on selles uuringus osalemisest saadav võimalik kasu?
Uuringus osalemine ei pruugi Teile otsest kasu tuua. Sellegipoolest võivad uuringu tulemused aidata leida uusi viise, kuidas tulevikus narkootikumide süstimist ennetada. Uuringu tulemused võivad aidata aru saada, kuidas pakkuda narkootikume kasutavatele inimestele paremini nõustamist ja ravi. Te saate tänu sekkumisele paremad teadmised, kuidas seista vastu survele ja vältida olukordi, mis võiksid viia esimese narkosüsti tegemiseni. Lisaks saate teavet ohutu süstimise ja üledooside ennetamise kohta. Peale selle toetatakse Teid juurdepääsul ennetus- ja tugiteenustele.

Kui otsustate uuringus mitte osaleda, ei mõjuta see Teie jaoks tavaliste ennetus- või raviteenuste kättesaadavust.

Millised on uuringuga seotud kulud?
Kõik uuringuga seotud visiidid, läbivaatused, laborianalüüsid ning muud protseduurid on Teile tasuta.

Millised on uuringuga seotud hüved?
Võimalike uuringus osalemisega seotud aja- ja reisikulude katteks antakse Teile igal visiidil toidupoe (Rimi) kinkekaart väärtuses 20 eurot ning järelvisiidil kupong väärtuses 20 eurot. Iga narkootikume mittesüstiva sõbra või tuttava eest (maksimaalselt 3 isikut), kelle olete uuringusse suunatud, saate pärast seda, kui nad on uuringus lõpuni osalenud, nende kaasamise eest uuringupreemia kinkekaardina (väärtuses 10 eurot).

Millised on Teie kohustused seoses uuringus osalemisega?
Andnud nõusoleku, olete võtnud kohustuse osaleda uuringutegevustes, mis põhinevad uuringukaval.

Kelle poole pöörduda küsimuste või probleemide korral?
Teie küsimustele selle uuringu kohta vastab Ave Talu, TÜ peremeditsiini ja rahvatervishoiu instituudi projektijuht (Lastekodu 3, Tallinn, telefon xxx xxxx).

Teie küsimustele oma õiguste kohta uuritavana saate vastuse, kui võtate ühendust Tartu Ülikooli inimuuringute eetika komiteega (Ülikooli 18, Tartu, sekretäri telefon 737 6215).

„Kombineeritud sekkumine süstimisega alustamise vähendamiseks"

Kui Te olete läbi lugenud käesoleva teadliku nõusoleku vormi või see on Teile ette loetud ja Te olete saanud vastused kõikidele oma küsimustele ning Te olete nõus uuringus osalema, kirjutage palun oma nimi, allkiri ning kuupäev dokumendi allolevale väljale.

Palun märkige ühte allpool olevatest lahtritest oma nimetähed, kinnitamaks, et uuringupersonal võib Teiega ühendust võtta näiteks olukorras, kus Teil jääb üks visiit vahele või kus Te ei saa uuringukeskusesse tulla.

**Jah, Te võite minuga ühendust võtta**	**…………**<br>(nimetähed)
**Ei, Te ei või minuga ühendust võtta**	..................<br>(nimetähed)

Palun märkide ühte allpool olevatest lahtritest oma nimetähed, kinnitamaks, et Teie loal sekkumine lindistatakse ja lindistused säilitatakse uuringu lõpuni.

**Jah, olen nõus**	..................<br>(nimetähed)
**Ei ole nõus**	..................<br>(nimetähed)

....<br>Uuritava nimi ja allkiri	………………………………<br>kuupäev / kuu / aasta
....<br>Uurija nimi ja allkiri	………………………………<br>kuupäev / kuu / aasta

„Комбинированные методы вмешательства для снижения числа первых попыток инъекционного употребления наркотиков»

ЛИСТОК ИНФОРМИРОВАННОГО И ОСОЗНАННОГО СОГЛАСИЯ ИССЛЕДУЕМОГО

Приглашаем Вас участвовать в научном исследовании, в ходе которого мы постараемся найти новые лучшие методы для замедления распространения инъекционной наркомании и ВИЧ (вирус иммунодефицита человека – *human immunodeficiency virus – вызывающий СПИД)* среди лиц, употребляющих наркотики.

Различные исследования показывают, что часть употребляющих наркотики всё же начинает наркотики колоть. У лиц, уколющих наркотики большая вероятность заразиться ВИЧ, чем у других групп лиц. Многие из нас никогда не тестировались на ВИЧ.

Также исследования показали, что потребители наркотиков иногда попадают в ситуацию, где им советуют или провоцируют их сделать первую инъекцию наркотика. Также могут люди, которые сами не колют наркотики, попасть в компанию, где расхваливают инъекционный способ употребления наркотиков, подчёркивая субъективные действия этого способа применения или же колются в присутствии других (не колющихся) людей. Кроме того, иногда колющиеся наркоманы помогают другим сделать свою первую инъекцию наркотика. Целью вмешательства данного исследования как раз и является разбор подобных ситуаций и желание предостеречь наркоманов от них.

Также различные исследования показали, что иногда колющиеся наркоманы одобрительно отзываются об инъекциях наркотиков, напирая на субъективные действия такого способа употребления или же колются в присутствии людей, которые не употребляют наркотики или сами не колются. Кроме того, колющиеся наркоманы иногда помогают другим людям в проведении их первой наркотической инъекции. Целью вмешательства является желание обсудить такие ситуации и предостеречь колющихся наркоманов от инъекций наркотиков другим.

Для чего проводится это исследование?
Исследование проводится для того, чтобы получить информацию о наркомании, связанной с инъекционным употреблением наркотиков. При этом сосредоточившись на первых попытках инъекционного употребления наркотиков или же отказа от этих попыток. Целью вмешательства, проводимого в рамках исследования, является необходимость предупредить причины первых попыток инъекционного употребления наркотиков. Будут рассмотрены также риски для человека, который решается на первую инъекцию наркотика. Так же в рамках вмешательства будут даны знания по безопасному проведению инъекций и предупреждению передозировки.

Всего в исследование будет вовлечено 300 лиц, употребляющих наркотики в течение последних двух месяцев, но не в виде инъекций, и которые живут в Таллинне и Харьюмаа. Приглашаем и Вас принять участие в данном исследовании.

Перед тем, как Вы решите принять участие в данном исследовании, Вы должны знать о возможных рисках, связанных с исследованием и пользе от него. Это называется процессом дачи информированного согласия. Данная форма информированного согласия даст Вам информацию о том, что будет с Вами происходить во время исследования и объяснит подробно обо всём, что связано с исследованием. В данном документе могут быть слова или термины, Вам незнакомые. Перед тем, как подписать форму согласия, попросите нас дать объяснения всего, что Вам непонятно. Если Вы подтвердите, что поняли смысл исследования и решите участвовать в нём, Вас попросят прочитать данную форму и подписать её.

В первую очередь очень важно, чтобы Вы знали следующее:
- Участие в исследовании добровольное

- Вы в любое время можете принять решение выйти из исследования
- Некоторые люди могут не подойти для участия в исследовании в процессе выяснения его пригодности на основании собранной информации

Что с Вами будет, если Вы решите участвовать в исследовании?

Выяснение пригодности для исследования

Вам зададут вопросы об употреблении наркотиков. Мы спросим Вас, проживаете ли в Таллинне или в его округе. Эта информация необходима, чтобы решить, годитесь ли Вы для участия в исследовании. В случае непригодности персонал центра может не объяснять Вам причины.

Вовлечение в исследование

Если Вы решите участвовать в исследовании, Вас уже сегодня включат в исследование и попросят явиться на повторный визит через 6 месяцев. На первом повторном визите Вы встретитесь с консультантом и второй повторный визит будет состоять из опроса и повторного взятия крови на тестирование её на ВИЧ и вирус гепатита С.

Первый визит (сегодняшний визит и продлится примерно 1,5 часа)

Во-первых, у Вас спросят о месте проживания и о других контактных данных, чтобы в будущем можно было бы связаться с Вами. Эта информация будет использована для того, чтобы напомнить Вам о времени последующего визита через 1 и через 6 месяцев после сегодняшнего визита. Мы спросим у Вас контактные данные Вашего доверенного лица (член семьи или друг), с которым мы сможем связаться, если нам потребуется найти Вас в связи с последующим визитом. Мы попросим Вас сообщить нам, какой способ контакта предпочитаете, если персонал исследования желает с Вами связаться. Персонал исследования объяснит Вам, какие методы предосторожности соблюдаются для обеспечения Вашей конфиденциальности. В конце этой формы у Вас спросят, разрешите ли Вы связаться с Вами.

Беседа с интервьюером продлится примерно 40 минут. Интервьюер спросит Вас о перенесенных ранее заболеваниях, сексуальной жизни и употреблении наркотиков. В связи с рискованным поведением Вам зададут вопросы об инъекциях наркотиков, наркомании, о лечении ВИЧ и передозировках. Также поинтересуются Вашим мнением по различным темам. Некоторые из задаваемых вопросов носят личный характер. Если Вы не желаете. можете не отвечать на них.

Во-вторых, Вас попросят принять участие во вмешательстве (в консультациях), основной целью которого является уменьшить вероятность того, что вы подтолкнёте кого-либо к инъекционному употреблению наркотиков или же сами начнёте колоть наркотики. Вам посоветуют, как избежать ситуации, способствующие переходу к инъекционному употреблению наркотиков. Также поделятся с Вами познаниями в вопросах по безопасным инъекциям и предупреждению передозировки. Консультация займёт минут 40. С Вашего позволения беседа будет записана на диктофон и записи будут сохранятся до конца исследования.

В-третьих, Вас попросят сдать 10 мл крови. Анализ крови будет проверен на наличие антител на ВИЧ и гепатита С. Ознакомление с результатами теста и после-тестовая консультация будут проводится во время первого повторного визита.

В-четвёртых, Вас ознакомят сегодня при желании со списком организаций, предлагающих в Таллинне необходимые услуги по профилактике и здравоохранению. К примеру, если Ваш тест на ВИЧ окажется положительным, то мы направим Вас в Инфекционный центр Ляэне-

Таллиннской Центральной больницы. Кроме того, Вам объяснят, как защитить себя и своего полового партнёра от ВИЧ и других венерических заболеваний, передающихся половым путём. Если желаете, то сотрудники исследования помогут Вам договориться об участии на обучающих курсах по профилактике передозировки.
Также с Вами договорятся о времени проведения сессии очередного повторного визита.

Участника исследования, который не колет наркотики или имеет небольшой опыт инъекционного употребления наркотиков, попросят вернуться на повторный визит через 1 месяц, чтобы встретиться с консультантом и обсудить с ним, были ли у Вас за прошедший месяц ситуации, которые могли бы спровоцировать переход на инъекционное употребление. Также выяснят, какой опыт Вы при этом прибрели и какой прок был от знаний, полученных на консультации.

Второй повторный визит через 6 месяцев
Вас попросят через 6 месяцев явиться на повторный визит. В ходе него будет проведён новый опрос, где кроме всего прочего мы спросим у Вас, каков оказался Ваш опыт в ситуациях, которые могли бы спровоцировать переход на инъекционное употребление наркотиков. Кроме того, у Вас возьмут 10 мл крови для определения антител на ВИЧ и гепатит С.
В случае, если Вы не явитесь на последующий визит, то персонал исследовательского центра, соответственно полученному Вашему разрешению, постарается для согласования нового времени визита отыскать Вас или Ваше доверенное лицо. К этому человеку мы обратимся в виде согласовонна с Вами сегодня. Мы обратимся к нему/ей чтобы напоминать Вам повторный визит или утачнить Ваши новые контактные данные.

С какими возможными рисками Вы можете столкнуться во время участия в исследовании?
Некоторые люди испытывают неловкость, когда им задают интимные вопросы. Запомните, что Вы не обязаны отвечать ни на какие вопросы, если не желаете этого. Мы гарантируем конфиденциальность данной Вами информации.
Из рисков, связанных со взятием крови, бывают обморок, боль и/или провоподтёк в месте взятия крови.. Процедуру взятия крови будет проводить обученная квалифицированная медсестра. Если результаты анализов покажут, что у Вас ВИЧ или гепатит С, то осознание этого может вызвать чувство подавленности.
Методы, которые члены исследовательской команды применяют для обеспечивания Вашей конфиденциальности, описаны ниже.

Как защищена Ваша конфиденциальность? Что делают с доверенной нам Вами информацией?

С целью защиты конфиденциальности Вам для участия в исследовании будет присвоен номерной код. Вопросник исследования, сопроводительное направление анализов в лабораторию и материалы, связанные со вмешательством (в том числе и записи на диктофоне), не содержит Вашего имени. Полученная от Вас информация будет введена в электронную базу данных под защитным кодовым замком, причём вместе с данными не будет храниться информация, позволяющая раскрыть Вашу личность. Собранные данные будут хранится в запертом сейфе и к ним имеют доступ только работники, причастные к проведению исследования.

Данные, собранные в ходе исследования, будут храниться бессрочно и будут использоваться в дальнейших научных исследованиях и связываться с другими источниками данных.

Вас нельзя будет вычислить по имени ни в одной отчёте или публикации. С Вашими исследуемыми данными смогут ознакомиться представители спонсора (Национальный институт Здравоохранения США).

На проведение данного исследования получено разрешение комитета по этике Тартуского Университета и команда исследования прошла основательное обучение.

Данные, полученные в ходе исследования будут сохранены и использованы только в научных целях в дальнейших научных разработках.

Какова возможная польза от участия в данном исследовании?
Участие в исследовании может не принести Вам прямой пользы. Но результаты данного исследования могут помочь нам найти новые методы, как предотвратить первые попытки инъекционного употребления наркотиков в будущем. Результаты исследования могут помочь нам понять, как лучше предлагать консультирование и лечение лицам, употребляющих наркотики. Благодаря вмешательству Вы получите лучшие знания, как противостоять напору и избежать ситуации, которые могут спровоцировать на первую инъекцию наркотиков. Кроме этого Вас поддержат в получении существующих профилактических и опорных услуг.

Если решите не участвовать в исследовании, то это не повлияет на получение Вами обычных профилактических и медицинских услуг по лечению

Какие будут затраты, связанные с исследованием?
Все визиты, осмотры, лабораторные анализы и другие процедуры, связанные с исследованием, для Вас будут бесплатными.

Какие будут связанные с исследованием бонусы?
Для компенсации затраченного времени и возможных транспортных расходов для прибытия на исследование, в ходе каждого визита Вам будет выдана подарочная карточка для посещения продуктового магазина (Рими) стоимостью 20 Евро и подарочная карточка стоимостью 20 Евро на повторном визит. За каждого направленного Вами для участия в исследовании друга/знакомого, являющегося не колющимся наркоманом (максимально 3 человека), после их участия в исследовании Вы получите в виде премии подарочную карточку (стоимостью 10 Евро).

Каковы Ваши обязанности, связанные с исследованием?
Участвуя в исследовании, Вы обязуетесь участвовать в исследовательских действиях соответственно плану исследования.

К кому Вы можете обратиться в случае возникновения вопросов или проблем?
На все Ваши вопросы по исследованию ответит Аве Талу, руководитель проекта института семейной медицины и общественного здравоохранения Тартуского университета (Ластекоду 3, Таллинн, телефон xxx xxxx)

На Ваши вопросы, касающиеся Ваших прав как исследуемого, можете обратиться в комитет Тартуского Университета по этике исследований человека (Юликооли 18, Тарту, телефон секретаря 737 6215)

„Комбинированные методы вмешательства для снижения числа первых попыток инъекционного употребления наркотиков“

Если Вы прочитали данную форму информированного согласия или Вам зачитали её вслух, и Вы получили ответы на все вопросы, и Вы согласны принять участие в исследовании, напишите, пожалуйста, своё имя, подпись и дату на нижеследующей строке:

Проставьте в одной из предложенных ниже графе свои инициалы, подтверждая тем, что персонал исследования может связаться с Вами, например, в случаях, когда Вы пропустили визит или Вы не можете прийти в исследовательский центр.

Да, вы можете связываться со мной .... (инициалы)

Нет, вы не должны связываться со мной .... (инициалы)

Проставьте в одной из предложенных ниже графе свои инициалы, подтверждая тем, что Вы согласны, что беседа будет записана на диктофон и запись будет сохранена до конца исследования.

Да, согласен/а .... (инициалы)

Нет, не согласен/а .... (инициалы)

.... ……………………………….
Имя и подпись участника исследования день / месяц / год

.... ……………………
Имя и подпись исследователя день / месяц / год

Lisa 11.5 Uuringus kasutatavad infovoldikud on välja töötatud Tervise arengu instituudi poolt.

1. HIV ja selle testimine. TAI 2017 (eesti ja vene keeles) (https://intra.tai.ee//images/prints/documents/148836164534_HIV_ja_selle_testimine_est.pdf; https://intra.tai.ee//images/prints/documents/148836167096_HIV-ja-selle-testimine_rus.pdf)
2. Ohutum süstimine infomaterjal süstivatele. TAI 2011 (narkomaanidelehttps://intra.tai.ee//images/prints/documents/131772254059_Ohutum_Systimine_EST.pdf)
3. Naloksoon. Elupäästja opioidi üledoosi korral. TAI 2016 (eesti ja vene keeles) (https://intra.tai.ee//images/prints/documents/144724980530_Naloksoon_EST_trykk.pdf; https://intra.tai.ee//images/prints/documents/14472498523_Naloksoon_RUS_trykk.pdf)
4. Kuidas tegutseda üledoosi korral? TAI 2016 (eesti ja vene keeles) (https://intra.tai.ee//images/prints/documents/144724994192_Kuidas_tegutseda_EST_trykk.pdf; https://intra.tai.ee//images/prints/documents/144724998580_Kuidas_tegutseda_RUS_trykk.pdf)

Lisa 11.6 Uuringus osalemise võimaluse tutvustav kutse.
Kutsume Teid osalema Tartu Ülikooli peremeditsiini ja rahvatervishoiu instituudi uuringus

Uuringud on näidanud, et mõned narkootikumide tarvitajad võivad erinevatel põhjustel alustada ka narkootikumide süstimist.

Narkootikume tarvitavad inimesed, kes ise ei süsti võivad sattuda seltskonda, kus räägitakse tunnustavalt narkootikumide süstimisest, rõhutades selle kasutamise viisi subjektiivseid tugevusi või süstitakse mitte-süstijate inimeste juuresolekul.

Varasematest uuringutest on selgunud, et narkootikumide kasutajad võivad sattuda olukordadesse, kus soovitatakse esimese narkootikumi süsti tegemist või pakutakse koguni abi selle tegemisel.

Tartu Ülikooli teadlaste uuringu eesmärgiks ongi selliseid olukordi arutada ning anda teadmised ja oskused sellistes olukordades käitumiseks.

2018-2019. aastatell läbi viidava uuringu täisnimetus on “ Kombineeritud sekkumine süstimisega alustamise vähendamiseks ” ja selle eesmärgiks on ennetada narkootikumidega süstimise alustamist ja sellega seonduvat (sh HIVi levimus, üledoosid jms).

Uuringus kasutatava nõustamise tõhusust ja ohutust on juba uuritud kõrge sissetulekuga riikides. Meie eesmärk on kohandada see sekkumine Tallinna ja Harjumaa olustikule.

Ootame uuringusse osalema Tallinnas ja Harjumaal elavaid täiskasvanud inimesi, kes tarvitavad narkootikume (näiteks amfetamiini tüüpi stimulandid, fentanüül ja kokaiin), kuid pole neid kunagi süstinud.

Kui Te soovite osaleda uuringus või soovite täpsemat teavet selle kohta, siis palun võtke ühendust projektijuhi Ave Talu telefonil 53 555 922 või e-mailiga uuring@ut.ee.
