# Supplementary material for: Adapted “Break the Cycle for Avant Garde” intervention to reduce injection assisting and promoting behaviours in people who inject drugs in Tallinn, Estonia: A pre- post trial
Source: PLoS One. 2023 May 31;18(5):e0266815. doi: 10.1371/journal.pone.0266815 (PMC10231841; doi:10.1371/journal.pone.0266815)
Supplement: S2 Protocol — (PDF) [file pone.0266815.s003.pdf]

RESEARCH STATEMENT FOR APPROVAL OF THE ETHICS COMMITTEE ON HUMAN RESEARCH AT THE UNIVERSITY OF TARTU

Combined prevention for reducing initiation into injecting drug use.

Sponsored by: National Institutes of Health

NIH Funding Mechanism: Grant #1DP1DA039542

Protocol Chair:

Don Des Jarlais, PhD
Beth Israel Medical Center
160 Water Street, Rm 2462
New York NY 10038
Phone +1 212 256 2548
Fax +1 212 256 2570
ddesjarlais@chpnet.org

Study Sites:

University of Tartu
Department of Public Health
Ravila 19, Tartu 50411, Estonia

NGO Convictus
Syringe exchange program
Lastekodu 6, Tallinn 10113, Estonia

Protocol Co-Chair:

Anneli Uusküla, MD, MS, PhD
University of Tartu, Department of Public health
Ravila 19, Tartu 50411, Estonia
Phone + 3727374195
Fax + 3727374192
Email anneli.uuskula@ut.ee

Study Coordinator:

Ave Talu, MS
University of Tartu, Department of Public health
Ravila 19, Tartu 50411, Estonia
Phone + 3727374195
Fax + 3727374192
Email marsh.kristina@gmail.com

Statistician
Mait Raag, MS
University of Tartu, Department of Public health
Ravila 19, Tartu 50411, Estonia
Phone + 3727374201
Fax + 3727374192
Email mait.raag@ut.ee

1. DATA ON THE RESEARCH

Full title of the research: Combined intervention to reduce the onset of injection.

In English: Combined prevention for reducing initiation into injecting drug use.

2. RESEACH LOCATION

Institute of Family Medicine and Public Health, UT, Ravila 19, Tartu 50411

MTÜ Convictus, Lastekodu 6, Tallinn 10115

3. RESEARCH PERFORMERS AND RESEARCH CENTERS

3.1. Responsible researcher

first and last name: Anneli Uusküla

degree: MD, MS, PhD

position: professor

job: University of Tartu, Institute of Family Medicine and Public Health, University of Tartu

job address: Ravila 19, Tartu 50409

phone number: 737 4195

e-mail: anneli.uuskula@ut .ee

date: signature:

3.2. Employees

first and last name: Ave Talu

degree: MSc

position: project manager

job: Institute of Family Medicine and Public Health, UT

job address: Lastekodu 3, Tallinn 10115

e-mail: ave.talu@gmail.com

date: signature:

first and last name: Greete Org

position: supervisor

job: Institute of Family Medicine and Public Health, UT

job address: Lastekodu 6, Tallinn 10115

e-mail: greete.org@mail.ee

date: signature:

3.3. The head of the institution of the responsible researcher or his or her acting person agrees to the organization of the research:

first and last name: Ruth Kalda

Position: Head of the Institute of Family Medicine and Public Health

Work place: University of Tartu

Work address: Ravila 19, Tartu 50409

Date: signature:

3.4. Approval of other institution involved in the research:

first name and surname of the authorized person: Inna Faber

Position: CEO

place of employment: MTÜ Convictus Eesti

work address: Lastekodu 6, Tallinn, 10113

date: signature:

4. BRIEF OVERVIEW OF RESEARCH CARRIED OUT ON THE SAME TOPIC

It is estimated that about 1% (5362 people; UI 3906– 9837) of the Estonian population aged 15–44 are injecting drug users (Uusküla et al., 2013). It has been found that injecting drugs is started at a very young age in Estonia. 23% of injecting drug users surveyed in Estonia started using drugs at the age of 15 and among them the prevalence of HIV was higher than among those who started injecting at a later age (48% versus 68%) (Vorobjov et al., 2013). Studies of injecting drug users in Estonia have shown that half of injectors have started using drugs immediately by injecting, and the other half of injectors have used drugs in some other way (oral / nasal, smoking, etc.) before starting injecting (Vorobjov 2012; 2013).

Registration of new HIV cases among injecting drug users has decreased in most EU Member States, but Estonia continues to be among the countries where a quarter to a third of new HIV cases (Estonia 25%, Luxembourg 27%, Latvia 32% and Lithuania 34%) are registered among the people who inject drugs (EMCDDA, 2017).

Efforts have been made to reduce drug use, injecting and injecting by informing people about the dangers of drugs, through legislative measures (punishment for users - fines, imprisonment) and extensive police

operations, in addition to removing drugs from the drug market and long-term drug trafficking offenses. Drug-related offenses (§ 151 of the NPLAS, ie the use, manufacture, acquisition or possession of a small amount of a narcotic substance) have risen sharply (in 2014 – 2862; in 2015 – 3633; and in 2016 - 4372) as the police's priority in recent years has been for drug users targeted activities (NIHD, 2017). The average fine imposed on drug addicts in Estonia for a drug offense (§ 151 NPLAS) is approximately 150 euros, plus expert examination costs.

Injecting drug use (sharing a used syringe) is a major cause of HIV and hepatitis C epidemics worldwide. It is also a major cause of other morbidity (non-fatal overdose, suicide attempts, skin and soft tissue infections) and mortality. Iintimidation and over-criminalization by law enforcement agencies have been found not to work and alternative strategies are needed (Godlee F., 2018)

In Portugal, drug use decriminalization reduced drug-related stigma, drug use and drug-related deaths and increased access to counseling, treatment and rehabilitation services (McCaffrey, 2009) and reduced drug-related health and non-health costs by 18%. (Gonçalves et al., 2015). In 2018, there is also a perception among various Estonian agencies (incl. the Ministry of Justice, the Ministry of the Interior, the Ministry of Social Affairs, NIHD, etc.) that paying addictions and procedural costs is too much for the people who use drugs and "treating" drug addiction with fines not working.

Interventions that prevent or delay injecting drug use are important, but there is little evidence in this area. Drug injecting prevention interventions can be targeted at: (i) injecting drug users; or (ii) non-injecting drug users and people with little injecting experience who use drugs other than by injection.

Only a few interventions have been tested and proven to be effective in preventing injecting drug use (systematic review: Werb D et al., 2013; Werb et al., 2018). It has been documented that some interventions, such as Break the Cycle (Hunt N et al., 1998) and the Heroin Sniffers Project (Heroin Sniffers Project) (Des Jarlais et al., 1996), are effective in preventing injection drugs use initiation. All of these interventions include a social networking and social support component and aim to change social norms related to injecting.

Injecting illegal drugs is a complicated and often dangerous procedure, and almost everyone who starts injecting needs the help of an experienced injector for their first injection. Injectors with long-term injecting experience are an important target group for prevention, as they have the skills and often the opportunity to assist those who have not previously injected with their first drug injection.

In study, conducted in Tallinn among PWID in 2016, 46% of the participants were infected with HIV, 66% of them were on antiretroviral treatment (Uusküla et al., 2018). One-fifth (20%) of those surveyed had been asked to help with their first injection in the last 6 months. 14% of the participants helped someone to inject them for the first time (4.3% had done so in the last 6 months); In the last six months, 9% of respondents had spoken positively about injecting drug use (better and faster effects and more cost-effective), 16% had injected themselves in front of a non-injecting person and 1.0% offered non-injecting help for the first time (Uusküla et al., 2018). Non-injecting drug use for the first time was more common in men (AOR 6.31, 95% CI 2.02–19.74), younger (30 years and younger) (AOR 3.89, 95% CI 1.40–10.16) and those with friends / acquaintances who had also assisted others in having an injecting drug for the first time (AOR 3.44.95% CI 1.31-9.03) (Uusküla et al., 2018). The last time the injectors were helped with the first drug injection, they were acquaintances (42%), friends (35%), sexual partners (13%) or strangers (8%). The main reason for assisting a non-injector with the first drug injection was the lack of skills to inject for the first time (78%), his condition (too nervous and shaking his hands) not allowing it (46%) or asking to inject himself (44%). For non-injecting injecting, they shared their drug with the injecting (33%), paid for it (2%) and 21% of the injectors wanted to share their experience (Uusküla et al., 2018).

According to recent studies in other developed countries, between 14% (Hamida et al., 2018) and 47% (Bluthenthal et al., 2014) of injecting drug users had helped some non-injectors to inject drugs for the first time in their lifetime. A study in Canada showed that in the lifetime, injectors had helped an average of 16 people inject drugs for the first time (Bluedenthal et al, 2014). A meta-analysis based on qualitative studies (n = 41) suggests that injecting has a unique meaning in humans and is associated with social and structural factors, so in addition to changing the behavior of interventions, the above factors should be addressed (Guise et al., 2017).

The transition from non-injecting to injecting drug use is not unavoidable. Various individual and social networking factors have been described that contribute to this (homelessness, unemployment, younger start-up of each opioid use, presence of acquaintances / friends / sexual partners of injecting drug users, support for injecting drug use by known friends or acquaintances, lower injecting drug use) (Germade M et al., 2007).

5. PURPOSE, SUMMARY AND JUSTIFICATION OF THE PROPOSED RESEARCH

The main objective of the study is to reduce initiation with injecting drugs by working in parallel to two target groups: (1) injecting drug users and (2) non-injecting drug users.

The study uses interventions based on motivational interviewing (MI).
Motivational interviewing is a client-centered, semi-directive method for enhancing intrinsic motivation to change problem behaviors by exploring and resolving ambivalence (Miller WR, 2002).

(1) MI has demonstrated effectiveness in promoting behavior change among persons with HIV and has been utilized to target co-occurring risk behaviors. Published results document that MI has the potential to reduce sexual (Naar-King S. 2012) and injection risk behaviors (Robles RR et al., 2004);

(2) MI is well adapted for IDUs' resistance to change and their difficulties related with involvement in long-term therapeutic process;

(3) Because MI is a method of communication, it can be utilized in many formats (e.g., single session or multiple sessions). Several single session MI interventions aiming on co-occurring risk behaviors reductions have been evaluated (Aharonovich E et al., 2012; Gilbert P et al., 2008);

(4) MI may be used alone to increase motivation for change or in combination with skills-building treatments, and many types of providers may deliver it.

MI is specified by its relational and technical components. Relational components refer to the counselor’s demonstration of empathy, collaboration, evocation and autonomy support, while technical components refer to specific techniques (e.g., use of open-ended questions, affirmations, reflections, summaries) to elicit and reinforce client verbalizations of arguments for change (“change talk”). The four general principles of motivational interviewing are: expressing empathy, developing discrepancy, rolling with resistance, and supporting self-efficacy (Miller WR, 2002).
Training in MI is particularly appropriate for the syringe exchange outreach staff because they will be able to use MI to support a wide range of health-promoting behaviors, including the use of the four primary interventions and the three other enhancements. We expect that the syringe exchange outreach workers will use motivational interviewing skills when interacting with subjects throughout the follow up period.

Motivational interviewing in the project:

(i) “Break the Chain” for injecting drug users to discuss how it has affected their lives, their first injecting experience and helping someone with their first drug injection and other activities

(demonstration, advocacy) which may affect someone starting to inject, and talk about the dangers of injecting, situations that could lead to someone injecting, sharing information with other injectors, and the dangers of helping others inject. In addition, the intervention introduces safe injection and addresses the risks of overdose and how to prevent them.

(ii) The 'Avoid the Needle', which advises current non-injecting drug users (who are at high risk of switching to injecting drug users, amphetamines and cocaine users) to identify situations that could lead to their first injecting drug, to avoid such situations and strategies to deal with them. The “Avoid Needle” also gives the participant a better understanding of safe injection and overdose prevention (Intervention instructions included to the Application).

Both interventions implemented in the study are based on the scientific literature presented below, have been adapted for use in Tallinn, and take into account local circumstances (most commonly used drugs, treatment and prevention options).

1) Des Jarlais, DC, Casriel, C., Friedman, SR, & Rosenblum, A. AIDS and the transition to illicit drug injection - results of a randomized trial prevention program. British Journal of Addiction 1992; 87: 493-498.

2) Casriel C, Des Jarlais DC, Rodriguez R, Friedman SR, Stepherson B, Khuri E. Working with heroin sniffers: clinical issues in preventing drug injection. J Subst Abuse Treat. 1990; 7 (1): 1-10.

3) Hunt, N., Stillwell, G., Taylor, C., Griffiths, P., 1998. Evaluation of a brief intervention to prevent initiation into injecting. Drugs Educ. Prev. Policy 5, 185–194.

4) Strike C, Rotondi M, Kolla G, Roy É, Rotondi NK, Rudzinski K, Balian R, Guimond T, Penn R, Silver RB, Millson M, Sirois K, Altenberg J, Hunt N. Interrupting the social processes linked with initiation of injection drug use: results from a pilot study. Drug Alcohol Depend. 2014 Apr 1; 137: 48-54.

5) Werb D, Buxton J, Shoveller J, Richardson C, Rowell G, Wood E. Interventions to prevent the initiation of injection drug use: A systematic review. Drug Alcohol Depend. 2013, Dec 133 (2): 669-676.

The adaptation of the interventions used in the study has been done in collaboration with researchers from New York, Icahn School of Medicine at Mount Sinai, and Columbia University. Don Des Jarlais is a researcher who designed and tested the Heroin Sniffer Project. Susan Tross, a practicing clinical psychologist.

Planned interventions have the potential to reduce injecting drug use, associated HIV infection, and reduce other injecting risks to the individual and society. The intervention implemented in the study also supports the prevention of overdoses and drug-related deaths, as it also includes the targeting of

preventive overdoses as a component. As part of the planned study, there is a good opportunity to inform addicts about the possibilities of professional counseling, drug treatment and harm reduction services (including the naloxone program for the prevention of overdoses and drug-related deaths). Usually, many people who have not yet become aware of the problems associated with their use and are not involved in counseling, treatment or other services are included in the study using a research-led engagement method. Research creates opportunities to direct them to the right services for them.

The main aim of the study is to reduce initiation with injecting drugs. We expect that the intervention will result in a reduction in injecting initiation and / or activity among participants.

6. TIME OF THE RESEARCH

The work is planned to be carried out (recruitment, follow up, data collection, testing for HIV and hepatitis C) during the period August 2018 to October 2019.

The recruitment period is approximately 6 months, during which a total of 300 drug users (both who inject drugs as well as non-injecting drug users and / or drug users with no previous injecting experience in the last 2 months) from Tallinn and Harju County will recruited.

For the study participant, the study lasts a total of 26 weeks, from the inclusion visit and the date of signing the informed consent to the second visit, during which time the participant visits the study center for two (injectors participating in the "Break the Chain" intervention) or three (non-injectors and / or ex-PWID who has not injected in the last 2 months participating in an "Avoid Needle" intervention).

Data entry, input control, and analysis will begin immediately after the subjects first participate in the study in August 2018.

Place of recruitment:

Recruitment of subjects for the study takes place in Tallinn in premises rented for the UT study (MTÜ Convictus Eesti, Lastekodu 6). The premises of MTÜ Convictus, which provides harm reduction services, are suitable for conducting the survey - with a central location in Tallinn, with a separate entrance both from the yard and from the street; a spacious reception room and two private rooms for interviews and interventions, and a special separate room for testing HIV and hepatitis C in private subjects.

Prior to conducting the research, the following training will be conducted: training

1. for the team involved in the study and data collection on the course of the study, the privacy of the individual in the study, and the confidentiality of the data;
2. interviewing training for survey interviewers;
3. HIV and hepatitis C testing (pre-test and post-test counseling) training at Synlab;
4. Counseling training for study participants (counselors):
    (i) training in intervention methodology - motivational interviewing (by EMITA - http://www.emita.ee/);
    (ii) intervention-specific training - trainers D. Des Jarlais, D. Barnes (to be held in July 2018)
5. study participants regularly participate in EMITA supervision during the study.

Participants in the study work as advisors to the target group in their day-to-day work and have participated as participants in previous studies described above. Interviewers who participated in the survey have also participated in conducting these surveys and have received prior training in interviewing.

7. STUDY SUBJECTS AND THEIR RECRUITMENT

In this study, the respondents are recruited using the respondent-driven sampling (RDS) method, which has been used in previous surveys of Estonian drug users (2005–2017). RDS is a validated method for accessing and recruiting people who use drugs (Malekinejad et al, 2008).

According to the study-led enrollment method, the first subjects to be selected in this study are 10 drug users (so-called “seeds”). In the course of the study, the “seeds” are first interviewed and then they are each asked to find three new subjects who meet the criteria of the study. To this end, the "seeds" are given three coupons with information about the study, including the RDS coupon number, the telephone number of the study center, e-mail, and the opening hours and location of the study. "Seed" gives the three coupons it receives to the next drug users known to it (injectors or non-injectors).

The activities related to the involvement of the subjects (incl. Clarification of the suitability for the study, clarification of the study procedures, informed consent, registration for the study, informing about the dates of the study visits) are performed by the supervisor (G. Org) or the project manager (A. Talu).

Criteria for inclusion in the study:

- a person aged 18 and older living in Tallinn and Harju County
- able to give informed consent to participate in the study

- has reportedly used illicit drugs (either fentanyl or other opiates, amphetamine, methamphetamine, ecstacy as the main drug used, except marijuana) in the last 2 months
- speaks Estonian or Russian,
- provide his contact details and agree that he may be contacted by the staff of the Research Center to recall the time of the follow-up meeting and the follow-up visit
- agrees to participate in the first main visit of the study, the follow-up visit (after 1 month) and the second main visit (6 months after the first main visit).

Exclusion criteria:

- participation in another HIV prevention or drug intervention study
- according to the assessment staff (disorder) condition (including alcohol or intoxication) or cognitive impairment that does not allow the subject to give informed consent or to follow the study visit schedule and study procedures.

The study visits will take place in the premises rented at the time of the study in Tallinn (MTÜ Convictus Eesti, Lastekodu 6, Tallinn), where there are suitable premises for private interviews of the subjects, planned intervention (counseling) and safe storage of the contact details of the subjects. There are 2 iron safes for storing research data, which can only be accessed by the research project manager, supervisor and the lead researcher.

Prior to initiating study-related procedures, the subject shall be thoroughly explained about the purpose, nature, benefits and potential harm of the study, including the intervention, and the measures taken to ensure the subject's confidentiality and secure contact information. The subject signs the subject's information and informed consent form (attached to the research application).

This follow-up study collects their personal data (first and last name, personal identification number, residential address, e-mail and telephone number) and the contact details of up to two close people (family member, friend, acquaintance) in case the subject cannot be reached in person, for example because that he frequently changes his telephone number and place of residence or does not have a fixed place of residence or does not use a telephone).

The subject who signs the information and informed consent form hereby consents to participate in the study (including the intervention), to be tested for HIV / HCV, and to retain and use his or her contact information for subsequent study visits.

If necessary, to determine if the subject is: (1) a non-injecting drug user, their skin will be screened for traces of injection and asked to describe in detail the preparation for use of the main illicit drug; or (2) the

person injecting the drug is checked for signs of injection in their skin and asked to describe in detail the preparation for and use of the illicit drug being injected as the main drug.

Storage and handling

The data collected in the study will be stored in an electronic database after entry (RedCap). The data of the subjects are entered using the impersonal code of the subject. The survey database, which contains questionnaire data and information on the test results and the subject's assessment of the intervention, does not contain information that allows the subject to be identified. The personal data of the subjects collected in the study are stored separately from the study data in an electronically password-protected file. The completed questionnaires, the subject's assessment of the intervention and the study monitoring forms on paper are kept in a locked cupboard for five years (in the premises of the Institute of Family Medicine and Public Health, University of Tartu, Ravila 19, Tartu 50411).

The data of the survey are entered and processed only at the Institute of Family Medicine and Public Health of the University of Tartu.

The data collected in the study will be used for scientific research purposes only. The data collected in the study will also be used in future research.

The Institute of Family Medicine and Public Health of the University of Tartu has developed a document entitled “Guidelines for Research Planning and Data Processing based on the requirements of personal data protection for the Institute of Family Medicine and Public Health of the University of Tartu.

8. DETAILED DESCRIPTION OF THE RESEARCH METHODS

BASELINE VISIT

Operations

1. Procedure for obtaining informed consent (the subject shall be thoroughly explained the purpose, nature, benefits and potential harm of the study and the measures to be taken to ensure the confidentiality of the study. The subject shall sign an informed consent sheet);
2. Record the participant's personal data (name / nickname, personal identification number, address, telephone number and e-mail address) in case he / she needs to be contacted in case of significant questions during the survey;
3. The subject is asked to provide the contact details (name / nickname, address, telephone number and e-mail address) of three of his / her close relatives (family members) and / or friends / acquaintances / e-mail, in a detention facility, in a medical institution due to illness, dead) can be obtained in connection with the investigation;

4. A structured interview will be conducted (questionnaires for injectors, non-injectors and injectors with little injection experience included in Estonian / Russian). In addition to demographic and socio-economic characteristics and the recruitment breakdown, the survey questionnaire includes selected questions on the age of drug and alcohol use and primary use, problems with main drug use, own drug use, exposure to injecting drug users (including receiving and / or assisting others in initial drug use) , on external norms related to the living environment, sexual risk behavior and drug-related risk behavior, overdose, psychological and physical health, harm reduction and drug addiction, ARV, access to hepatitis C treatment options and social support.

Based on the interview, the need for a specific intervention is identified:

i. Injecting drug users receive a 'break the chain' intervention

ii. Non-injecting drug users receive “Avoid the needle” intervention

5. It takes about 40 minutes to answer the questions; the interview is conducted in Estonian or Russian, depending on the subject's wishes. The questionnaires for the three groups of subjects (injectors, have little injection experience, never injected) differ on a small scale (injectors are also asked questions about injecting) (Appendices 11.3.1 and 11.3.2);

6. Subjects undergo pre-HIV and HCV counseling by a trained employee; and 15 ml of venous blood is collected from the test for the presence of antibodies to HIV and HCV. Venous blood is collected by a person trained by a nurse, following all the principles of infection control.

Testing for HIV / HCV antibodies is performed by SYNLAB Eesti OÜ (Veerenni 53a, 11313, Tallinn) IV generation ELISA method. The laboratory of SYNLAB Eesti OÜ is accredited by the Estonian Accreditation Center (accreditation certificate L159) and follows the standard ISO 15189 “Medical laboratories. Special Requirements for Quality and Competence ”and follows the legislation in force in the Republic of Estonia in organizing its activities. HIV positive tests in the screening test are verified by Western blot analysis (according to national rules). The results of the tests are communicated to the subject within 7 days of the test;

7. The supervisor of the study center will agree with the study participant on the exact time of the next visit.

Study Counseling (s)

The Study Intervention “Avoid the Needle” consists of two individual intervention (counseling) sessions conducted by the intervener. The duration of the first is ~ 60 minutes and the duration of the second (which takes place after 4 weeks) is about 20 minutes. Participants in this intervention have either never injected drugs or little experience with injecting, but in a different way in the last two months than the subjects using the injections.

Intervention instructions for injectors (Annex 11.4.1.3), never for injectors (Annex 11.4.1.1) and for persons with ex-injection experience (Annex 11.4.1.2) are provided as annexes to the application.

With the consent of the subject, the intervention sessions are recorded to *fidelity* of the intervention. In addition, the respondent is asked to complete a feedback form to evaluate the intervention.

FOLLOW UP VISIT

All subjects participate in the second main visit (6 months after the baseline visit).

<u>Operations</u>

1. A structured interview will be conducted with selected questions on injecting and assisting others, on sexual risk and drug-related risk behaviors under investigation, on criminal background and on access to drug treatment options. It takes about 45 minutes to answer the questions, the interview is conducted in Estonian or Russian according to the preference of the respondent;
2. If the test HIV and / or HCV test was negative at the previous visit, you will be asked to have a new blood test for antibodies to HIV / HCV at the second visit (6 months after the first main visit);
3. The results of the tests will be communicated to the subject as part of post-test counseling no later than 1 week after testing for HIV and hepatitis C. In the event of a positive test result, the subject will be referred to and / or informed and / or assisted by a gastroenterologist for hepatitis C treatment at an LTKH Infection Diseases Clinic.

PARTICIPANT RETENTION

Once a participant enrolls in the study, the study site will make every effort to retain him/her for the full study period 6 months to minimize possible bias associated with loss-to-follow-up. Retention rates of at least 80% at 6 months are targeted among participants who remain alive and are not incarcerated for the duration of the study.

Study site staff will be involved in developing and implementing necessary local standard operating procedures to target this goal.

Components of such procedures include:

• Thorough explanation of the study visit schedule and procedural requirements to the participant during the informed consent process; this will be re-emphasized at each study visit; Use of appointment cards to

remind participants of appointment dates; Collection of detailed locator information at the baseline visit (Phone number, Email address, Address, Phone and mailing address of a trusted person (preferably parent, husband/wife) as per approval of the study subject), Phone and email or mailing address of the trusted peer); Use of appropriate [as per agreement with the participant] and timely visit reminder mechanisms:

• Immediate and multifaceted follow-up on missed visits;

• Mobilization of trained outreach workers to complete in-person contact with participants at their homes and/or other community locations;

• Regular communication with the study community at large to increase awareness about HIV/AIDS and explain the purpose of HIV prevention research and the importance of completing research study visits.

The potential events related to illicit drug use that warrant monitoring (incarceration, death) and that cannot be obtained from study participants due to the losses for follow up will be elicited through contacts with named peers, family members if possible.

PARTICIPANT WITHDRAWAL

Participants may voluntarily withdraw from the study for any reason at any time. The site Investigator also may withdraw participants from the study to protect the safety and well-being of the participant and/or the study staff and only after consultation with the local principal investigator.

Every reasonable effort will be made to complete a final evaluation of participants who terminate from the study prior to the last scheduled follow-up visit. Study staff will record the reason(s) for all withdrawals from the study in participants’ study records

MISSED VISITS/LOSS TO FOLLOW-UP

Site research staff will make every effort to have participants complete the follow-up study visit. If a participant refuses any protocol-specified procedures, this will be appropriately documented in the study documents. In person contact with participants is the preferred method of collecting study data. However, there may be rare circumstances in which telephone contact) may be the only method available for collecting data. Determining that a telephone contact may be necessary, it will be done only after all other options for scheduling the visit have been explored (e.g., earlier or later office hours, interim visits). All scheduling efforts will be documented in the participant’s contact logs.

9. DESCRIPTION OF ETHICAL ASPECTS OF RESEARCH

The investigators will ensure that this study is conducted in full conformity with the current revision of the Declaration of Helsinki, or with the International Conference for Harmonization Good Clinical Practice (ICH-GCP) regulations and guidelines, whichever affords the greater protection to the subject The protocol, informed consent forms, participant recruitment materials, and other requested documents, and any subsequent modifications, will be reviewed and approved by the ethical review bodies responsible for oversight of research conducted at the University of Tartu, Estonia.

This is an experimental study in which subjects belong to a stigmatized *population* due to their drug use. It is very important to ensure the autonomy of the subjects, the voluntary nature of the participation in the study and the dignified and respectful treatment.

We have thoroughly analyzed the potential risks associated with participating in the study and are implementing appropriate and recognized measures to minimize them.

Potential damage related to the investigation

(1) loss of personal privacy;

(2) loss of confidentiality of collected data;

(3) physical discomfort in taking a blood sample;

(4) psychological discomfort in answering questions about drug use and sexual life;

(6) social harms related to participation in the study (eg stigma, discrimination based on drug status or sexual behavior).

The research staff is very experienced in working with this target group and has previously participated in the experimental intervention studies “Break the Chain” and “Avoid the Needle” among injecting drug users. The people working in the study (supervisor, project manager, interviewers, interveners) have also received the motivational interviewing training necessary to communicate with the target group, which ensures a dignified, respectful and respectful attitude towards the subjects. Regular supervisors of the participants and regular meetings of the entire research team to discuss and resolve research issues help to ensure a good working atmosphere.

Survey in private settings is a prerequisite for interviewing, performing interventions, and testing for HIV and hepatitis C.

The data collected in the survey are stored in an electronically password-protected file in the database with the coded survey results of the survey (answers to the interview questions, results of the analyzes, evaluation

of the intervention). Completed questionnaires and study monitoring forms on paper will be kept in a locked cabinet for five years.

To reduce potential psychological discomfort, study staff have received prior training in conducting the study and all subjects will be provided with contact information for the study coordinator and supervisor. Subjects may contact the coordinator with any questions regarding the study and / or drug use.

The information leaflet with the contact details of the centers dealing with drug reduction and HIV / AIDS counseling and prevention in Tallinn is also distributed to the respondents. Under both interventions, subjects will be provided with a leaflet on the prevention of overdoses of various drugs. In addition, they will be provided with the contact details of MTÜ Convictus and, at their request, the time of naloxone training will be agreed with them (once a week at MTÜ Convictus Eesti).

In order to minimize social harm, all measures shall be taken to protect the personal data of the subject and the information collected during the study. In case of psychological distress, specially trained research staff are always available to discuss the problems and find solutions, including referring the NGO Convictus to a free reception for a psychologist.

Subjects are offered counseling in the event of a positive HIV or hepatitis C test. The nurse of the study discusses with the subject the various options for seeking treatment and, if necessary, arranges a doctor's visit to the infection center.

Benefits of the study

The information gathered in the study will provide an opportunity to assess the feasibility and effectiveness of interventions to prevent and / or reduce injecting drug use and to further monitor the spread of HIV and hepatitis C in the affected population. The study will provide valuable information for the development of appropriate prevention measures and health and harm reduction services for the target group.

Benefits for the study participant

Participation in the study allows referral and sharing of information on injecting and non-injecting drug users (referral to rehabilitation or substitution treatment, outpatient and outpatient clinics, HIV treatment, social rehabilitation). In addition, information materials and free condoms are distributed to respondents.

10. DATA HANDLING AND RECORD KEEPING

Hereby we describe steps to be taken to assure that the data collected are accurate, consistent, complete and reliable and in accordance with ICH GCP guidelines.

Procedures:

Hardware:

Prior to the commencement of data collection, computer systems will be in place to manage the study data. Data Management staff will maintain independent personal computers. All computers will be configured for automatic security updates.

Physical Security:

Desktop computers used for the study will be located at study site (NGO Convictus) and at the Department of Public health, University of Tartu. Desktop computers will be password protected with unique passwords. The buildings are equipped with standard fire safety equipment.

Data Security:

All electronic data will be stored on desktop computers password-protected with unique user passwords. Electronic data will be backed up weekly and can be retrieved in the event of a problem. Data collected on paper forms will be stored in a locked file cabinet at Department of Public health, University of Tartu. Access to all data is restricted to study PIs, designated study personnel and the study sponsor.

Data Collection Tool Development:

Data collection paper forms will be developed by study coordinator and the PIs. The project data manager will perform routine weekly random spot-checks for all entered forms, assessing accuracy and completeness.

Data Completion Instructions:

Data collected using face-to-face interviews, study monitoring forms and laboratory forms will be entered twice using electronic data entry program (double data entry) into the computer by the data entry clerk, at the Department of Public health, University of Tartu.

All handwritten data collected on paper forms will be written clearly by data collection staff so that it can be read by other persons (for example, there should be a distinction between the number 5 and the letter S, when written).

Any errors will be corrected by a single line drawn through the incorrect data, with the date and initials of the staff member that made the correction.

Data Storage:

Storage space (filing cabinets) for paper forms will be locked at all times and maintained at SEP of NGO Convictus. Collected data forms will be transported from the SEP of NGO Convictus to Department of Public health, University of Tartu monthly in the course of the study.

Storage space (filing cabinets) for paper forms will be locked at all times and maintained at Department of Public health, University of Tartu, which is a secure facility.

All filing cabinets will be locked. Only the local PI and designated study staff will have keys to cabinets and closets. Informed Consents and participant locator information will be stored separately from data collected to ensure names are not linked to data collected. All data collected will be identifiable only by a unique ID number. The researchers at University of Tartu and Beth Israel Medical center will have access to this data.

Data Entry:

Data files will be exported to SAS, encrypted by the study statistician at Department of Public health, University of Tartu for report development when required by study sponsors or the Principal Investigator. Data files will not contain any identifying information.

Data from paper forms, such as participant locator forms, will be entered into a database on password-protected desktop computer by study staff on a weekly basis.

Data Error Correction:

Data received from data collection staff will be reviewed by the data manager for missing data, skip errors and the like. Any errors will be corrected. Old versions will be saved and archived. Corrections will be made and a new version created. The new version will have a new version number and a new date of creation. QDS automatically creates a 'change history' which can be accessed from its menu.

Database Archiving:

The databases (e.g., SAS) will be stored electronically on a password protected desktop computer to be used for further analysis when required. Final datasets will be stored on a password protected desktop computer that will be accessible to the Principal Investigator and study statistician, and other study staff at the Principal Investigator's discretion. Datasets will be used for analysis when required.

Record Retention:

Hard copies of records (e.g., consent forms, participant locator forms) will be stored in a locked data storage facility at Department of Public health, University of Tartu for five years after study completion and then destroyed. Electronic records (data files) which do not contain identifying information will be stored in a password protected secure location at Department of Public health, University of Tartu.

11. ANNEXES:

11.1 CV of the PI researcher and co-researchers

11.2 Forms of informing and informed consent of the subject (in Estonian and Russian)

11.3 Study survey instrument (in Estonian and Russian)

11.4 Intervention instructions for injectors (Annex 11.4.1.3), never for injectors (Annex 11.4.1.1) and for persons with ex-injection experience (Annex 11.4.1.2)
